# Perturbing Pentalene: Aromaticity and Antiaromaticity in a Non‐Alternant Polycyclic Aromatic Hydrocarbon and BN‐Heteroanalogues

**DOI:** 10.1002/cphc.202401069

**Published:** 2025-03-16

**Authors:** Cate S. Anstöter, Patrick W. Fowler

**Affiliations:** ^1^ EaStCHEM School of Chemistry University of Edinburgh Edinburgh United Kingdom; ^2^ School of Mathematical and Physical Science University of Sheffield Sheffield United Kingdom

**Keywords:** *Ab initio* calculations, aromaticity, antiaromaticity, ring currents, current-density maps, ipsocentric, symmetry, selection rules

## Abstract

Pentalene (C_8_H_6_) and NN‐ and BB‐bridged heterocyclic analogues (BN)_4_H_6_, derived by replacement of CC pairs with BN, are taken as paradigms for tuning of ring‐current (anti)aromaticity by variation of π charge, electronegativity and substitution pattern. *Ab initio* calculation of maps for the π current density induced in these model systems by a perpendicular external magnetic field exhibits the full range of tropicity, from diatropic aromatic to nonaromatic to paratropic antiaromatic, with a ready rationalisation in terms of an orbital model. Further calculations on systems of varying charge in which these motifs are embedded in extended PAH systems with naphthalene and phenanthrene ‘clamps’ show promise for switching between current patterns and related opto‐electronic properties. Particular sensitivity to charge is found for the experimentally accessible NN‐bridged heteropentalene hybrids.

## Graphical abstract

Substituting BN for CC pairs has dramatic consequences for magnetic response of a molecule. Pentalene, an antiaromatic paradigm, provides an interesting system in which wholesale replacement of CC with BN gives two minimally frustrated isosteres. Here, we map the effects of electronegativity, substitution pattern and charge on ring current of bare pentalene isosteres and synthetically isolated *π* extended systems.

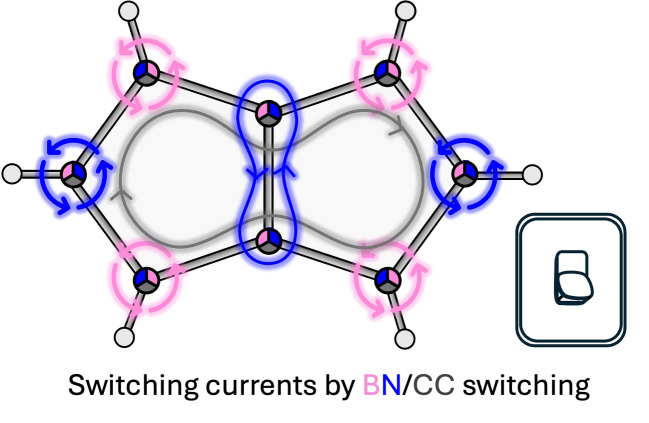

## 1 Introduction

Antiaromatic π systems are attracting increasing attention for their distinctive reactivity,[^1^, ^2^] low band gap,^[3]^ and redox switchability.^[4]^ These properties are of interest in multiple applications in, for example, optoelectronics,^[5]^ functional materials^[6]^ and solar cells.^[7]^ Given the tendency of antiaromatic systems to ‘escape’ to nonaromaticity *via* changes in geometry^[8]^ or electron count,^[9]^ incorporation into larger chemical frameworks can be an attractive option for capturing their intrinsic properties.[^3^, ^10^] Understanding of antiaromaticity has lagged behind the study of aromaticity, with the term itself being introduced in 1967 by Breslow,^[11]^ a century and a half after Faraday first isolated benzene.^[12]^

Presence of a ring‐current is a widely accepted criterion for the diagnosis of aromaticity and antiaromaticity.^[13]^ In a molecule that supports a ring current, the diatropic or paratropic circulation of π electrons induced by an external magnetic field is taken as definitive, with diatropicity corresponding to aromaticity and paratropicity to antiaromaticity.

The benzene molecule is clearly the paradigm for aromaticity on magnetic and many other criteria. It is less clear which molecule holds the equivalent position for antiaromaticity. One popular candidate is planar‐constrained cyclooctatetraene (COT). COT exhibits a strong paratropic ring current in a planar setting,[^14^, ^15^] but in the absence of symmetry or steric constraints ‘escapes’ *via* first‐order Jahn‐Teller distortion to the nonaromatic $D_{2d}$ tub‐shaped structure. In this equilibrium geometry of COT, π currents have localised and the ring current has vanished.^[16]^

Arguably, pentalene (**1**) is a better representative of antiaromaticity, in that it supports a paratropic ring current both in its highest possible $D_{2h}$ symmetry and at the $C_{2h}$ equilibrium geometry attained *via* second‐order Jahn‐Teller distortion.[^17^, ^18^, ^19^] Pentalene is a textbook example of a distortive π system.[^20^, ^21^, ^22^] The origin of its global paratropic ring current is well understood in terms of orbital contributions obeying symmetry‐based selection rules that generalise those for monocycles.[^14^, ^15^]

Pentalene was first synthesised as the dianion in a lithium salt.[^23^, ^24^] Neutral pentalene was obtained by matrix isolation of the photo‐cleaved dimer,^[25]^ and later synthesised in a protected form bearing three tert‐butyl substituents, for which the ^1^H NMR spectrum indicated the presence of paratropic ring current.^[26]^ A recent study successfully synthesised a tetraphenlypentalene, stabilising the antiaromatic pentalene core in the neutral form through sterics. Intriguingly, the reduction between the neutral and dianion state was demonstrated to be reversible, switching between antiaromatic (neutral) and aromatic (dianion) states.^[27]^ Pentalene has featured extensively in subsequent literature as a ligand in organometallic complexes, in various coordination modes with specific electronic requirements.[^28^, ^29^] Furthermore, this structural motif has been proposed as an important π ‐system for novel electronic materials^[30]^ and as a building block in ladder‐type π ‐conjugated molecules.^[31]^ The pentalene dication has apparently eluded synthesis so far, but has been encountered as a motif in a cationic polycyclic aromatic hydrocarbon.^[32]^ Calculations of ring current at the simple Hückel‐London level for the eight‐carbon framework^[33]^ indicate that both dication and dianion of pentalene should sustain *diatropic* perimeter π ring‐currents in place of the paratropic current of the neutral. These predictions are arguably in broad agreement with calculated NICS values.^[34]^ Again, this variation of tropicity with π electron count is consistent with the symmetry‐based selection rules derived from the ipsocentric approach to calculation of magnetic response.[^14^, ^15^]

In discussions of aromaticity it is traditional to compare isosteric systems with the same formal π electron count, such as benzene, triazine and borazine.^[35]^ Borazine has often been claimed to be ‘the inorganic benzene’, but that particular analogy does not survive scrutiny, as the π system supports localised lone‐pair π circulations rather than a global ring current.^[36]^ However, for the corresponding eight‐membered ring, ipsocentric calculations indicate that the *dianion* of B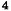 N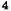 H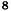 would support a fully fledged benzene‐like diatropic ring current.^[37]^

It seems natural to explore similar analogies for *antiaromatic* systems based on pentalene. There is an extensive chemistry of partially substituted heteropentalenes incorporating boron and nitrogen.^[38]^ Pentalene motifs with multiple substitutions have been targeted in synthesis for their potential as opto‐electronic materials,^[39]^ and it has been noted that changes to the precise sequence in heteroatom chains within the motif can have a decisive influence on their properties.^[5]^

**Figure 1:**
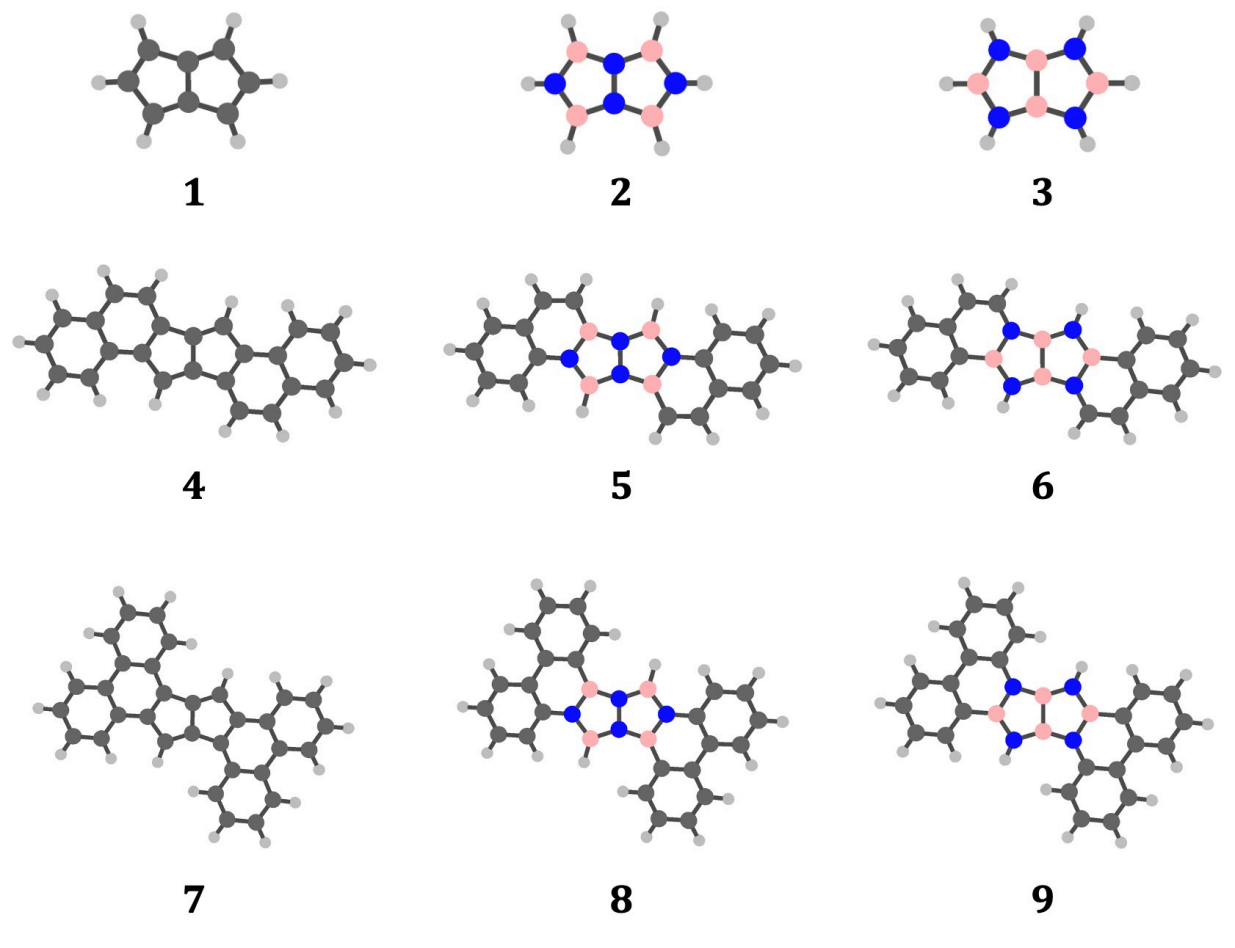
Molecular graphs of pentalene (**1**) and the two B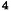 N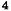 H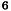 ‐isosteres with minimum edge frustration, NN‐frustrated **2** and BB‐frustrated **3** (top row). Extended π frameworks that incorporate (hetero)pentalenes are shown in the middle and bottom row. Systems **4**, **5** and **6** are created from naphthalene fragments with a central pentalene, while structures **7**, **8** and **9** are created by formal fusion of phenanthrene fragments. No specific geometry, point group or bonding scheme is implied. In the colour scheme for atoms, H is grey, C is charcoal, N is blue, and B is pink.

As the starting point for the present investigation, we consider the heteropentalenes where *all* CC pairs have been replaced by BN so as to achieve minimum frustration by homonuclear pairs. These isosteres are in the same relationship to borazocine (B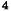 N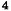 H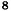 ) as pentalene is to COT, but have two isomeric forms, defined by whether the bridging bond is NN or BB (**2** and **3** in Figure 1). Structure **2** has been synthesised in protected form.[^39^, ^40^]

Using the model structures **1**–**3**, we investigate the effect of varying the charge and the substitution pattern on current‐density maps, as predictors of aromaticity, nonaromaticity or antiaromaticity. The analysis is then extended to larger π systems **5**–**9**, based on experimentally realised structures that incorporate pentalene isosteres.^[41]^ Design principles are suggested for producing extended hybrid PAH/BN systems that have a desired magnetotropicity.

Crucial to this process is a specific feature of the formulation of magnetic response *via* the *ipsocentric* approach,[^14^, ^15^] where current arises from well‐defined and physically realistic orbital contributions governed by symmetry selection rules. When combined with a simple electronegativity model, this approach gives a ready interpretation of trends in aromaticity and antiaromaticity in both isolated and extended pentalenes as a function of π electron count.

## Methods

All (hetero)pentalene and extended (hetero)pentalene geometries were optimised at the ω B97x‐D/aug‐cc‐pVDZ level of theory. Charge states (π ‐electron counts) treated in this way were **1** ^2+^(6π ), **1** ^0^(8π ), **1** ^2−^(10π ), **2** ^2+^(6π ), **2** ^0^(8π ), **2** ^2−^(10π ), **3** ^2+^(6π ), **3** ^0^(8π ), **3** ^2−^(10π ), **4** ^2+^(22π ), **4** ^0^(24π ), **4** ^2−^(26π ), **5** ^2+^(22π ), **5** 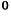 (24π ), **5** ^2−^(26π ), **6** ^2+^(22π ), **6** ^0^(24π ), **6** ^2−^(26π ), **7** ^2+^(30π ), **7** ^0^(32π ), **7** ^2−^(34π ), **8** ^2+^(30π ), **8** ^0^(32π ), **8** ^2−^(34π ), **9** ^2+^(30π ), **9** ^0^(32π ), **9** ^2−^(34π ), All structures were found to have planar minima as checked by diagonalisation of the Hessian at the given level of theory. Details of the point group symmetries of all species can be found in the ESI, along with cartesian coordinates and frequencies of low‐energy vibrational modes. These DFT calculations were performed using the Q‐Chem 6.1 computational package.^[42]^

Current‐density maps were calculated at the CHF/CTOCD‐DZ2/6‐31G** *ab initio* level with the Modena/Exeter/Sheffield version of the SYSMO package.^[43]^ A newer version is implemented as SYSMOIC.^[44]^ Despite the modest size of the 6‐31G** basis set, in combination with the distributed‐origin ipsocentric approach it delivers well‐converged currents.^[45]^ The combination of DFT geometries and CHF currents has been found to give excellent results for current maps, in particular for closed shells.^[46]^

In the following sections, we will show maps of total induced π current density and the most significant orbital contributions to them, for various species. The current‐density maps follow our established conventions: they show the first‐order induced current density (i. e. the first derivative of current density with respect to the magnetic field). For *ab initio* maps, the plotting plane lies 1 $a_{0}$ above the plane of the nuclei. Current‐density vectors are resolved onto the plotting plane, and the full modulus of current density is shown as an underlying contour map. Nuclear positions projected into the plotting plane are represented by colour‐filled circles, where H is white, C charcoal, B pink and N blue. The maximum magnitude of π ‐current density over the gridpoints in the plotting plane, *j* 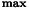 , is used as a measure of relative current strength; this quantity is interpreted with reference to the equivalent for benzene calculated at a height of 1 $a_{0}$ at the same level of theory.^[47]^

The minimum‐energy geometries of the neutral species **1**‐**9** were initially used to calculated the current‐density maps of each dication, neutral, and dianion, as a way of separating the effects of variation in π ‐electron count from those of structural changes. Later, for reasons outlined below, all maps for **1**‐**3** were also recalculated for the minimum‐energy structure appropriate to the charge state (see ESI). In fact, the sole case for which the constraint to the geometry of the neutral proved problematic was **2** 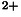 (see section 2.3 for a full discussion).

Table 1 lists the selection rules for diatropic and paratropic current[^14^, ^15^] as they apply to groups $D_{2h}$ and $C_{2h}$ in the setting where the molecule lies in the *xy* plane.

**Table 1:** Ipsocentric selection rules for excitations contributing to current in $D_{2h}$ and $C_{2h}$ symmetry. An entry * implies no current, $R_{z}$ implies paratropic, and x and y implies diatropic current.

	A 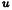	B 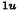	B 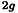	B 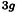
A 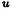	*	R 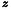	y	x
B 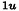	R 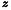	*	x	y
B 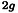	*	x	*	R 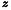
B 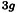	*	y	R 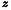	*
				

	A 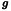	A 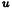	B 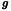	B 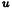
A 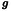	R 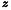	*	*	(x,y)
A 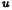	*	R 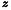	(x,y)	*
B 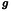	*	(x,y)	R 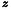	*
B 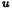	(x,y)	*	*	R 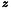

Broadly speaking, within the ipsocentric approach, a description in terms of canonical molecular orbitals (CMOs) with well‐defined symmetry within the point group will be the most economical in terms of the number of orbital contributions to be summed, and symmetry selection rules will be at their most powerful, for delocalised π systems. For fully localised π systems, however, localised molecular orbitals (LMOs) will provide the cleanest description.^[48]^ If we are to retain σ/π separability, the choice of localisation scheme is important; the particular scheme used here is due to Pipek and Mezey.^[49]^ Both types of analysis are needed for **1**–**3**, which crisscross the territory between the extremes of delocalised and localised behaviour.

As symmetry selection rules are modulated by energy considerations,^[15]^ the analysis for delocalised systems often turns out to be dominated by contributions from frontier orbitals. For monocycles this rationale aligns with a chemically intuitive understanding in which the Hückel 4n/4n+2 rule of stability resolves into a contrasting 2π /4π picture of the genesis of current.^[14]^

The use of modest basis sets for anions has known limitations. In the case of ring current calculations this tends to constrain the electronic configuration to valence molecular orbitals. We employ a relatively modest basis set in the ipsocentric ring‐current calculations for all charge states. Whilst this is an appropriate approach for neutral and cationic systems,^[45]^ it neglects the inherent susceptibility of small molecular (poly)anions to auto‐detachment.^[50]^ For dianions, it biases the electron attachment to occupation of $\pi^{*}$ molecular orbitals, rather than the pseudo‐continuum orbitals found with extended basis sets.^[51]^ Inspection of the electronic configuration of the dianions of the two heteropentalenes calculated with DFT revealed that the additional two electrons occupy a pseudo‐continuum orbital rather than a valence orbital, indicating metastability. The extended systems with embedded pentalene and heteropentalene motifs did not show this behaviour, as the the extra electrons occupy $\pi^{*}$ orbitals. To further specify the ground state wavefunction of the dianionic heteropentalenes, additional electronic structure calculations, using extended multi‐configurational quasi‐degenerate perturbation theory (XMCQDPT2),^[52]^ were run with the Firefly computational package.^[53]^ These calculations employed a (10,9) active space of eight π /$\pi^{*}$ orbitals and one highly diffuse p‐orbital, occupied by ten electrons. Population of the diffuse orbital is taken to occur at the threshold for neutral‐plus‐free‐electron(s) auto‐detachment, an assumption that has been demonstrated to be in excellent agreement with experimental studies on other systems.[^54^, ^55^] For heteropentalenes **2** 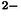 and **3** 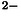 , the two extra electrons populate this orbital, confirming the thesis that the dianions are metastable, also at this level of theory. However, for our purpose of using **1** to **3** as models preparatory to their incorporation in larger systems, this is not a concern.

## 2 Results and Discussion

### 2.1 Neutral Pentalene and Heteropentalenes

**Figure 2:**
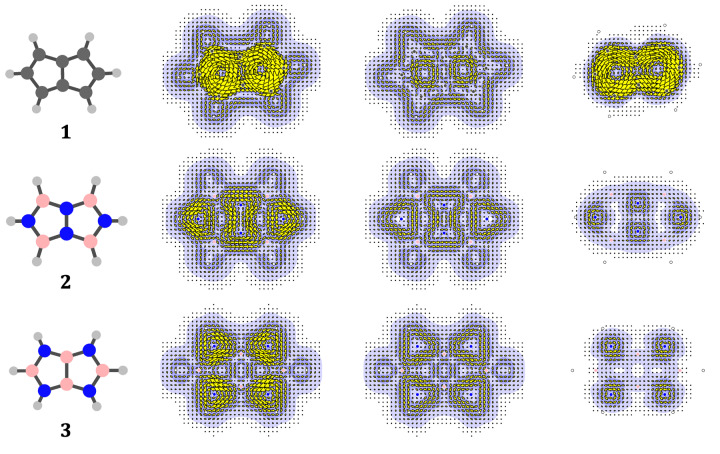
Current‐density maps for neutral pentalene and isosteres, **1** 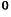 , **2** 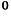 and **3** 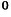 . The columns of maps show respectively, total current (σ + π ) and partitions into contributions from σ and π manifolds. Colouring in the maps follows that of the molecular graphs, where C is charcoal, B is pink and N is blue. All maps are computed for a height of 1a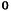 above the plane of the molecule, following the plotting conventions detailed in section Methods.

Current‐density maps of the paradigm species, pentalene **1** 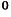 and isosteres **2** 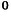 and **3** 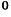 , are reported in Figure 2. The figure shows total (σ+π ), σ ‐only and π ‐only maps (all for a height of 1a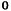 above the molecular plane). The maps of total (σ +π ) current give an immediate impression of the differences between systems: pentalene **1** 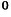 has a strong paratropic ring current, but NN‐bridged **2** 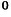 and BB‐bridged **3** 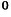 show a more ‘granular’ pattern. Resolution into σ and π contributions confirms that the global paratropic current in pentalene originates in the π system, and that the localised currents of both **2** 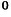 and **3** 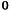 arise from a combination of contributions of the sp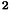 σ bonds, generating a local ‘triangular’ σ circulation, and p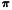 ‘lone‐pair’ circulations associated with the N centres.

The pure σ maps illustrate the behaviour expected of a closed‐shell molecule, with an outer diatropic current flowing around the exo CH (resp. BH, NH) bonds, superposition of local diatropic currents for framework two‐electron σ bonds, and resultant minor paratropic vortices at ring centres.^[56]^ The main message derived from the set of σ maps is of their smooth and uneventful nature, demonstrating the ability of the ipsocentric method to describe both (magnetically) localised and delocalised systems.

The π ‐current maps are more varied and show sharper distinctions between different species. For neutral pentalene, **1** 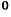 , the map shows a strong, global paratropic (antiaromatic) ring current.

In maps for species **2** 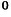 and **3** 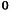 , the π current is a simple superposition of lone‐pair contributions from the four N atoms, as found for the unbridged neutral borazocine.^[36]^ In terms of π ‐tropicity, **1** 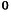 is an antiaromatic, whereas **2** 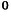 and **3** 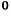 are nonaromatic.

As will be discussed in detail below, the delocalised ring current of neutral pentalene is consistent with simple Hückel and ipsocentric pictures, in which the dominant HOMO‐LUMO excitation occurs within a split rotational pair and hence leads to paratropic current, as in planar‐constrained COT. In the latter case, the pair is split by Jahn‐Teller distortion from $D_{8h}$ to $D_{4h}$ symmetry; in the case of pentalene it is already split by the introduction of a chordal σ bond. Currents across split rotational pairs tend to be stronger than diatropic currents, for well understood reasons. Here, the paratropic π current in pentalene is stronger than the oppositely directed π ring current of benzene and the paratropic current of planarised COT, calculated at the same level, by factors of 1.5 and 2, respectively.

In contrast, the localised π ‐current maps for **2** 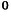 and **3** 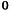 are not attributable to decisive contributions from specific frontier canonical orbitals (CMOs), as the HOMO‐LUMO gap is widened by electronegativity effects, and the current in these cases is more efficiently described as contributions from four individual localised molecular orbitals (LMOs). The parallel with delocalised benzene and localised borazine current maps^[48]^ is apparent.

### 2.2 Pentalene and Heteropentalene Ions

**Figure 3:**
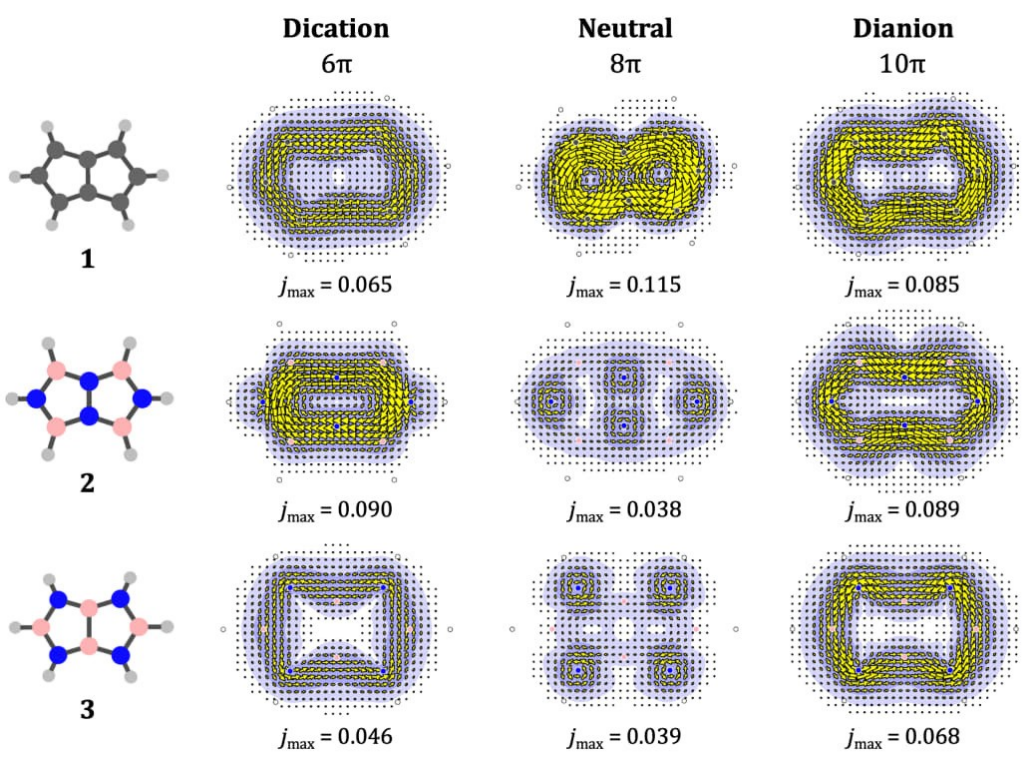
Current‐density maps for pentalene and isosteres in charge states with different π electron counts. The three rows show the π ‐current calculated for the 6π dication, 8π neutral and 10π dianion of **1**, **2** or **3**, respectively. In each case the geometry of the corresponding neutral species was used. Plotting conventions are as in Figure 2. Values *j* 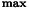 indicate the maximum in the plotting plan of the induced current per unit field (in a.u.). For reference, the value *j* 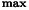 calculated for benzene at the same level of theory is 0.078 a.u., and for COT 0.515 a.u.

We now consider the effect of total charge on the π currents in **1**, **2** and **3**. To isolate the pure charge effect, we begin by making maps for the ions at the frozen geometry of the respective neutral system. Figure 3 compares the π currents in pentalene and the two heteropentalenes computed in this way as functions of total charge and π ‐electron count. As noted earlier, our choice of basis set ensures that the difference in total charge is reflected in π populations. For pentalene, the strong paratropic π current of the 8π neutral is replaced by (weaker) diatropic ring currents around the 8‐cycle perimeter. These predictions for the all‐carbon framework are also consistent with the purely graph‐theoretical version of Hückel‐London theory.^1^ For NN‐bridged **2** and BB‐bridged **3**, the changes are more dramatic, in that each switches from localised π currents in the neutral to delocalised perimeter π ring currents in the ions. There is a marked dependence on the bridge atoms: for NN **2** 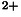 (at the frozen geometry of the neutral) and **2** 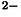 , the global π ring currents are paratropic in the dication and diatropic in the dianion; for BB **3** 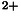 and **3** 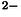 the global π ring current is diatropic in both cases.

On pure considerations of electron count, the switch from the localised π currents of the neutral heteropentalenes to delocalised π currents in the ions is consistent with the simplest model of pentalene as an 8‐cycle perturbed by a central chordal edge. The 10π count of **2** 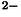 and **3** 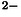 equates to filling of the former HOMO‐LUMO pair of the perturbed COT cycle, thereby shutting off rotational excitations within the |Λ|=2 pair, and opening up the translational excitations from it to the |Λ|=3 pair, to yield perimeter diatropic current. Likewise, the 8π count of **2** 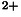 and **3** 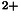 leaves the former HOMO‐LUMO pair empty, allowing translational excitations from |Λ|=1 to |Λ|=2 , again consistent with perimeter diatropic current. Quenching of delocalised current in the neutrals **2** ^0^ and **3** ^0^ is consistent with opening the gap between occupied and empty shells, as in neutral borazocine.^[36]^

The only difficulty for this simple interpretation is the calculated global *paratropic* current for **2** 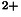 , the NN‐bridged dicationic heteropentalene. To investigate this case we need a more careful account of the dependence of electronic structure on the placing of heteroatoms around the COT‐like 8‐cycle and the unavoidable frustrated BB or NN pair across the chordal edge. To discuss this dependence, we use a model that includes the different electronegativities of N and B explicitly.

### 2.3 Extended Walsh Diagrams for Analysis of Currents

Qualitative conclusions about molecular geometry can be derived from correlation diagrams for orbital energies plotted against geometric parameters such as the classic Walsh diagrams for molecular hydrides.^[57]^ A similar idea, where the ordinate represents electronegativity difference, has been used for BN‐substituted hydrocarbons.[^36^, ^37^, ^58^, ^59^] For a qualitative model of the effect of electronegativity alternation around the molecular perimeter of pentalene, we consider a weighted graph in which N vertices carry a weight of +ηβ and B carry -ηβ , where β is the Hückel resonance parameter and the term η represents the opposite electronegative/electropositive characters of N and B. Use of a vertex‐weighted graph with equal and opposite weights gives rise to particularly simple expressions for the energy levels of a π system,^[60]^ showing how they are affected by electronegativity difference.

In the Hückel model, orbital energies are given by the roots of the characteristic polynomial (the eigenvalues of the weighted adjacency matrix). For COT, the characteristic equation is

(1) $$\left(\mathrm{COT}\right)\;\lambda^{2}{\left(\lambda^{2}-2\right)}^{2}\left(\lambda^{2}-4\right)=0,$$

yielding the (1,2,2,2,1) pattern of degeneracies of the 8‐cycle. In pentalene, this pattern is broken by the intervention of a σ bond as a diameter, and the new equation is

(2) $$\left(\mathrm{pentalene}\right)\;\lambda \left(\lambda -1\right)\left(\lambda +2\right)\left(\lambda^{2}-2\right)\left(\lambda^{3}-\lambda^{2}-4\lambda +2\right)=0$$

This is further modified in the case of the NN‐bridged heteropentalene to

(3) $$\left(\mathrm{NN}\right)\;\left(\lambda +\eta \right)\left(\lambda^{2}-\left(\eta^{2}+2\right)\right)\left(\lambda^{2}+\lambda -\eta \left(\eta -1\right)\right)(\lambda^{3}+\left(\eta -1\right)\lambda^{2}-\left(\eta^{2}+4\right)\lambda +\left(\eta^{3}+\eta^{2}+4\eta +2\right))=0,$$

and the characteristic equation for BB‐bridged heteropentalene follows by changing the sign of η :

(4) $$\left(\mathrm{BB}\right)\;\left(\lambda -\eta \right)\left(\lambda^{2}-\left(\eta^{2}+2\right)\right)\left(\lambda^{2}+\lambda -\eta \left(\eta +1\right)\right)(\lambda^{3}-\left(\eta +1\right)\lambda^{2}-\left(\eta^{2}+4\right)\lambda -\left(\eta^{3}-\eta^{2}+4\eta -2\right))=0.$$

**Figure 4:**
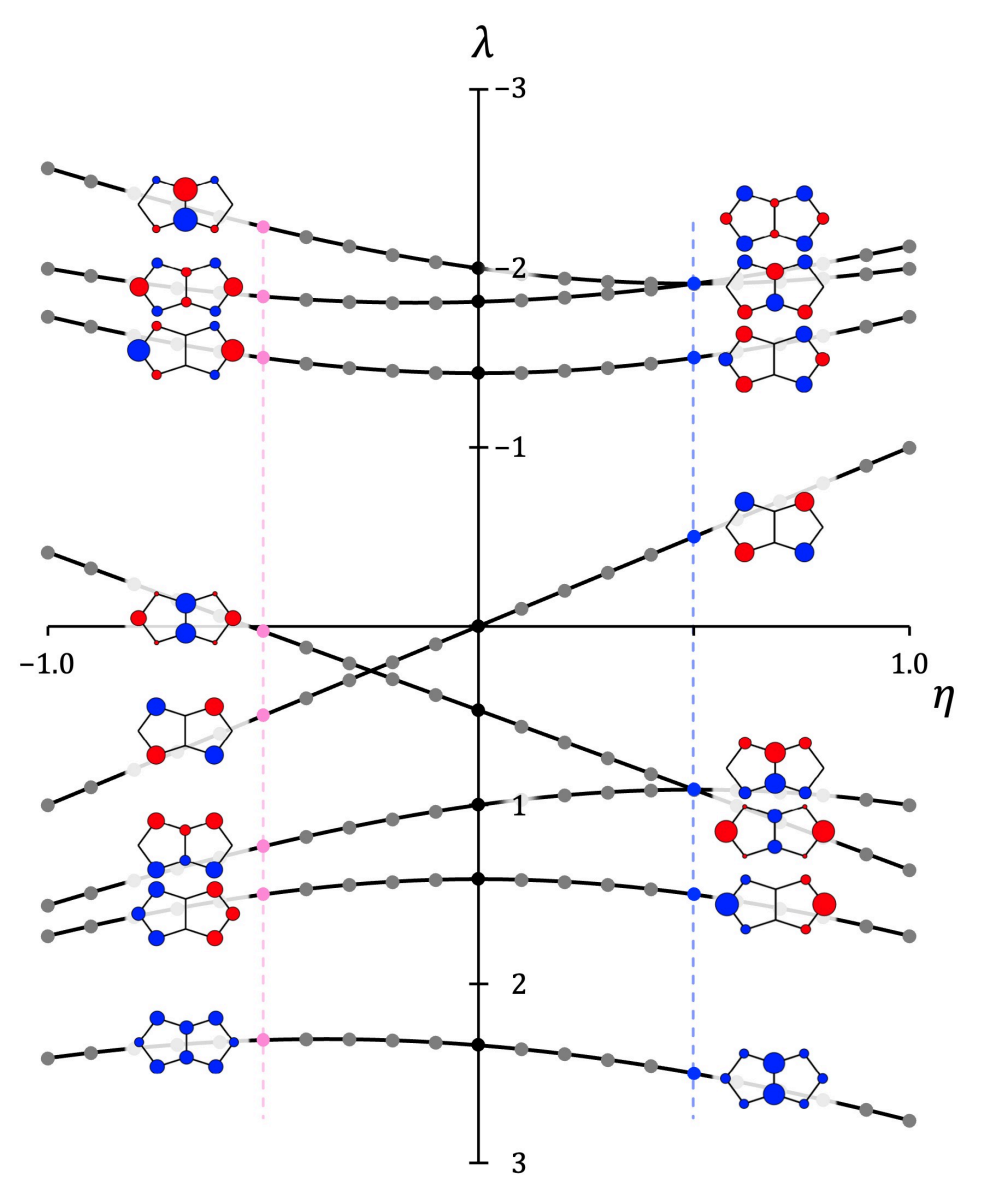
Schematic correlation diagram showing the variation with electronegativity of Hückel π molecular orbitals and orbital energies in B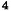 N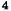 H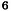 heteropentalenes. Eigenvalues λ are shown on the vertical axis; the horizontal axis shows the electronegativity of the central atom pair (both axes in units of β ). The central portion of the diagram represents pentalene **1**; the righthand side represents NN‐bridged species **2** and the lefthand side the BB‐bridged species **3**. Eigenvalues are plotted as filled circles, with curves drawn to guide the eye. Black, blue and pink circles denote eigenvalues for **1**, **2**, **3**, with nominal choices $\eta =0,+\frac{1}{2},-\frac{1}{2}$ . Inset diagrams show Hückel eigenvectors for $\eta =\pm \frac{1}{2}$ , with circle radii and colours indicating magnitude and relative sign of eigenvalue entries.

Figure 4 presents an extended Walsh‐diagram showing molecular orbital energies derived from Eqns. 3 and 4 as functions of the electronegativity parameter η , within this simplified model of heteropentalenes. where the distinction between B and N is captured by taking a single compromise value of |η| . Two obvious features of this plot are the strong curvature of the highest and lowest MO energies and strict linearity of energies of the HOMO‐LUMO |Λ|=2 pair. The diagram predicts a reordering of the π manifold from **3** 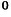 to **1** 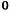 to **2** 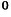 . As |η| moves away from zero, density shifts onto electronegative centres in the bonding MO and off electropositive centres, and *vice versa* for the antibonding manifold.

A further significant feature of the diagram is the existence of crossing curves on either side of η=0 . The lower crossing at positive η is potentially significant for the NN‐bridged dication and needs further analysis. At the crossing at η=0.5 , Λ=1 and Λ=2 orbitals (both occupied in the neutral) become degenerate, and so for η>0.5 the dication would correspond to removal of a π pair from an MO of Λ=1 , leading to two rotationally allowed transitions and an unusual 4π *paratropic* current (see Figure 3). In contrast, for η<0.5 the gap would correspond to a translational transition and *diatropic* current, which points to a complicating factor for precisely the system that has anomalous behaviour on the crudest Hückel model, i. e. **2** 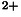 .

**Figure 5:**
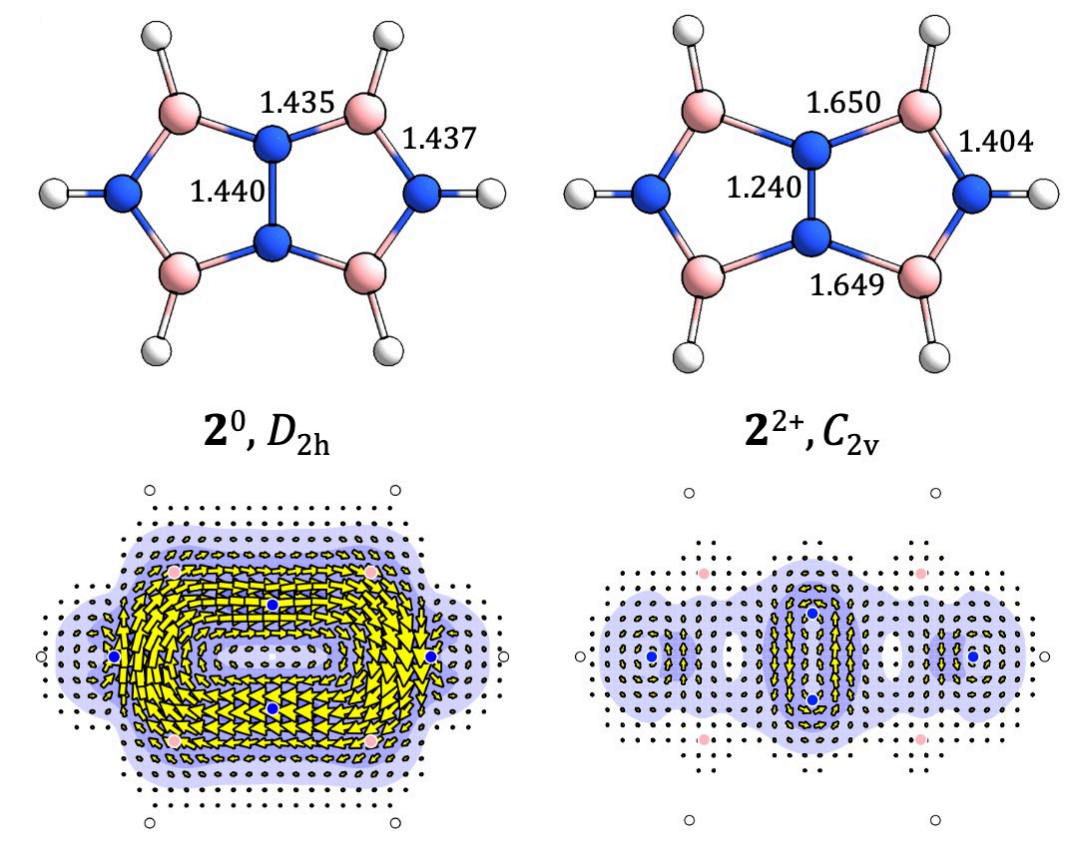
Influence of geometric relaxation on the total π currents for a dicationic heteropentalene. **2** 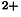 in the unrelaxed $D_{2h}$ geometry of the neutral species **2** 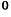 (bottom left) shows a strong paratropic perimeter current. In the relaxed $C_{2v}$ geometry (bottom right) the currents are localised, with a dominant *diatropic* circulation on the central N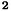 moiety. This is consistent with the major changes in framework bond lengths (shown in Å) between the two geometries (top row).

To check the sensitivity of the delocalised current in **2** 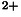 to the assumed geometry used in the calculation of the maps of Figure 5, the geometric constraint was relaxed. Full optimisation of **2** 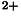 leads to a $C_{2v}$ structure with a much shorter NN contact (1.204 Å) corresponding to a double bond N=N, compared with 1.440 Å in the $D_{2h}$ geometry of the neutral corresponding to a single bond N−N). The π current map computed for the relaxed geometry is now very different in appearance, with localised ‘lone‐pair’ diatropic circulations on the isolated N centres and a diatropic bond current on the central NN bridge, accounted for by the sum of three spatially separated π LMO contributions (see Figure S2).

Geometries and π current maps for the frozen and relaxed structures of **2** 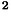 are shown in Figure S3. Checks for the six charged species **1** 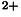 , **1** 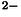 , **2** 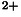 , **2** 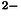 , **3** 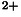 , and **3** 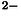 , (see section Methods) showed that the π map for the NN‐bridged dication is the only one to show a qualitative change in current pattern on full optimisation (see Figure 5). The example of **2** 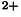 is a salutary reminder that we cannot expect to get a correct account of current by using a geometry that implies an incorrect electronic structure.^[46]^

### 2.4 The *Ipsocentric* Account of Current Maps of Charged Heteropentalenes

**Figure 6:**
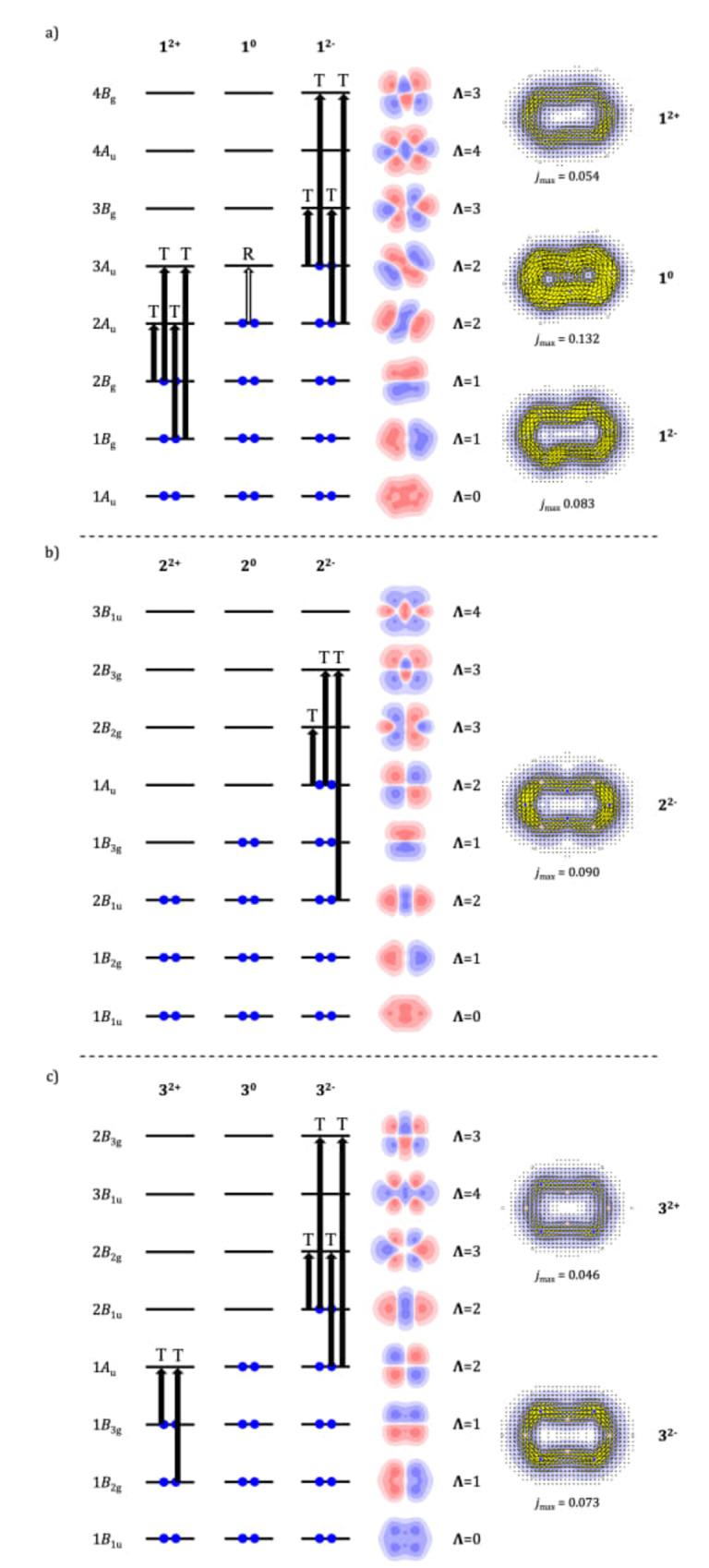
Orbital analysis of π ‐current maps for pentalene and heteropentalenes with different π counts. Panels a), b) and c) show the energy ordering (not to scale) and nodal structure of canonical π molecular orbitals for species $\left\{1^{2+},\;1^{0},\;1^{2-}\right\}$ , $\left\{2^{2+},\;2^{0},\;2^{2-}\right\}$ and $\left\{3^{2+},\;3^{0},\;3^{2-}\right\}$ and the symmetry labels of orbitals in the symmetry of the respective neutral parent, $C_{2h}$ (**1** 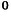 ) and $D_{2h}$ (**2** 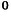 and **3** 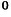 ). Full/hollow arrows represent dominant virtual excitations allowed under translational and rotational selection rules (leading to diatropic and paratropic current). The maps are constructed by summation of the dominant orbital contributions. Transitions for neutrals $2^{0}$ , $2^{2+}$ and $3^{0}$ are not shown, as these are localised systems at their respective equilibrium geometries.

Finally, we confirm that the maps for charge states that exhibit delocalised π current are indeed consistent with the ipsocentric frontier‐orbital selection rules. Figure 6 shows the dominant orbital contributions for those species that have delocalised magnetic response. Predictions of global paratropic ring current (for **1** 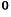 ) and global diatropic ring current (for **1** 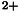 , **1** 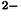 , **2** 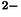 , **3** 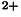 , **3** 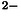 ) are consistent in sense and strength with the accessible occupied‐to‐empty virtual excitations from HOMO and HOMO‐1 in all cases. The shifts from translational to rotational character follow the changes in occupied‐virtual symmetry products as orbitals are progressively added to the occupied list and removed from the list of low‐lying virtuals. The sense of each orbital contribution can be rationalised by considering the node counts and angular‐momentum classifications, also illustrated in the figure.

To reprise: we begin with neutral pentalene, **1** 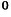 Here, the dominant orbital contribution is from the HOMO (2*A* 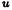 ) and this is turn is dominated by the rotationally allowed HOMO‐LUMO excitation, which can be computed as described previously.^[61]^ In this respect, pentalene follows the classic 2π antiaromaticity previously observed for COT. In **1** 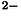 , this excitation is blocked by occupation of the second member of the Jahn‐Teller rotational pair. In the cation **1** 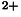 , both Jahn‐Teller orbitals become available as targets for virtual excitations. In both cases currents follow in the simplified Hückel model of COT.

For both NN‐ and BB‐frustrated isosteres, we can apply a similar orbital analysis to the ion maps, for which global currents are observed. For **2** 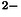 , we note that current is dominated by four π electrons (pair to pair transitions), one orbital contribution from Λ=2 , 1*A* 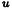 to the next Λ=3 pair, and the lower lying Λ=2 , 2*B* 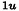 . Interestingly, the Λ=1 , 1*B* 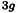 HOMO‐1 of **2** 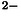 is magnetically dark, as it has no available Λ+1 transitions owing to occupancy of both Λ=2 CMOs. The case of **2** 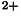 is also consistent with the selection rules, once account is taken of geometry dependence. If the frozen **2** 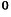 geometry is used for the cation, there are two major contributions from rotationally allowed transitions between Λ+1 CMOs, giving the unusual 4π paratropic current. If instead the relaxed **2** 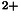 structure is used, the π current localises on isolated N centres and the NN bridge, and is seen to arise from superposition of three π LMOs (see Figure S2).

The neutral system, **2** 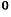 , has essentially localised lone pair currents in the π ‐map (see ESI). Analysis using CMOs recovers this pattern, but at the expense of including contributions from all four occupied π orbitals, whereas switching to the LMO representation gives a one‐to‐one association between the MO localised on a given N centre and the lone‐pair circulation that it supports (see Figure S1). Precisely similar observations can be made for charge states of isostere **3**.

The model heteropentalenes exhibit a variety of magnetic behaviour, and in particular indicate strong dependence on geometric structure in nitrogen bridged heteropentalene. It remains to check whether these features change when the model species are embedded in larger ‘clamped’ macrostructures.

### 2.5 Extended Pentalenes and Heteropentalenes

Embedding of a motif such as pentalene in an extended π system raises natural questions about persistence or quenching of its ring current, generation of new macrocyclic currents, and connections between these effects and total charge.

Our choice of systems for exploring the interaction of heteropentalene motifs with a PAH‐like environment is guided by recent synthetic work on neutral molecules with an embedded NN‐bridge heteropentalene by Kashida *et al*.^[41]^ An assessment of magnetic response of **5** 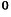 and **8** 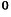 was made in that paper using NICS calculations. The motivation for the study was the possibility of interesting new properties emerging from the combination of π systems, with specific reference to applications in organic light‐emitting diodes. Figure 1 shows structures made by formal clamping of motifs **1**–**3**, between pairs of naphthalene and phenanthrene moeities, to form systems **4**–**9**. The structures **5** and **8** correspond to the synthesised molecules.^[41]^

In what follows, we examine charge states −2, 0 and +2 for all six frameworks, and compute π current density maps as a means of characterising magnetic response and evaluating the implications of clamping and charge for aromaticity/antiaromaticity. We note that there has been historic intrigue around the dibenzopentalenes and their unique redox behaviour.^[31]^ It turns out that optimised structures for the ions of **5** do not show the drastic changes in NN bond length with total charge that were found for the isolated motif **2** (optimised NN bridge distances in **5** 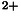 , **5** 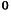 and **5** 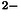 are resp. 1.470 Å, 1.437 Å and 1.440 Å, all consistent with an N−N single bond on the bridge). Calculation of the π current maps for the clamped systems was therefore carried with frozen neutral geometries in order to isolate pure charging effects. Maps of σ current were also constructed (see Figure S4) but showed no significance dependence on total charge, as expected, since σ populations do not change between charge states.

**Figure 7:**
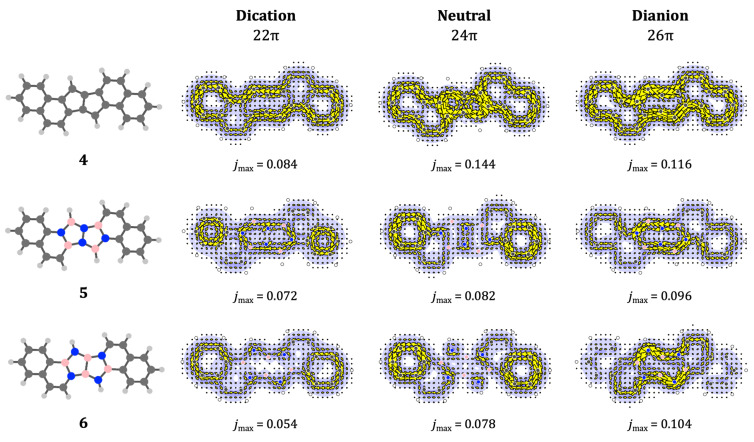
π density maps for the three naphthalene‐extended pentalene isosteres, **4**, **5** and **6**. Maps for dicationic, neutral and dianionic charge states are shown, i. e. the 22π , 24π and 26π species. All maps are computed at the ($C_{2h}$ ) minimum‐energy structure of the respective neutral isostere. Plotting, colouring conventions and definition of *j* 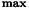 are as in previous figures.

To establish a baseline for comparison we begin with the all‐carbon frameworks. The π maps shown in the first row of Figure 7 for the naphthalene‐clamped **4** exhibit current flow over the whole framework. At the 24π (neutral) electron count the antiaromatic JT‐signature current of bare pentalene survives, as an island trapped between the two characteristic diatropic circulations of the naphthalene clamps. In the charged systems 22π (dication) and 26π (dianion), the diatropic currents expected for the charged pentalenes merge smoothly into a concerted diatropic flow around the perimeter, which is typical of fused PAH systems.

Moving to the heteropentalene motifs, it was found that in the case of NN‐bridging the enlarged systems again retained π circulations characteristic of the central motif. For neutral 24π and dianion 26π counts, the patterns in **5** are dominated by lone‐pair currents and diatropic circulations on the 8‐cycle, respectively. In the case of the dianionic species **5** 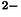 the extra electron pair does indeed enter the π system at all levels of theory (see section Methods).

For the 22π NN‐bridged **5** 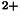 , we see a delocalised current on the central motif but observe that it is *not* the paratropic current found for the bare motif with the central N- N single bond, but rather it is *diatropic*. This reiterates the magnetic versatility of the NN‐pentalene, which can be aromatic, nonaromatic or antiaromatic, according to charge and environment. A tentative explanation of the ‘escape from antiaromaticity’ of **5** 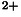 is that the frontier orbitals of the larger system show strong mixing of motif and clamp, changing the balance of excitations. Systematic effects of clamping on currents have been noted before for benzene and COT: native currents may be quenched or reversed on clamping by π systems, where similar geometry changes induced by saturated clamps leave them undisturbed.^[62]^

The non‐innocent nature of the clamping process may apply in both directions. For the heteropentalene systems **5** and **6**, the notional integrity of the formal naphthalene/phenanthrene units is disturbed by replacement of some of their atoms. This is accompanied by disruption of their characteristic perimeter currents, which is apparent in all charge states of **5** and **6**, where naphthalenoid 10‐cycle naphthalene currents break up into separate 6‐cycle circulations in the neutrals.

The most interesting case here is again the NN‐bridged dication. In this case we see two formally ‘antiaromatic’ benzene units and a central ‘diatropic’ motif. Given the synthetic interest in redox‐active aromatic switches,[^4^, ^63^] these dramatic reversals of current may be worth exploring further.

**Figure 8:**
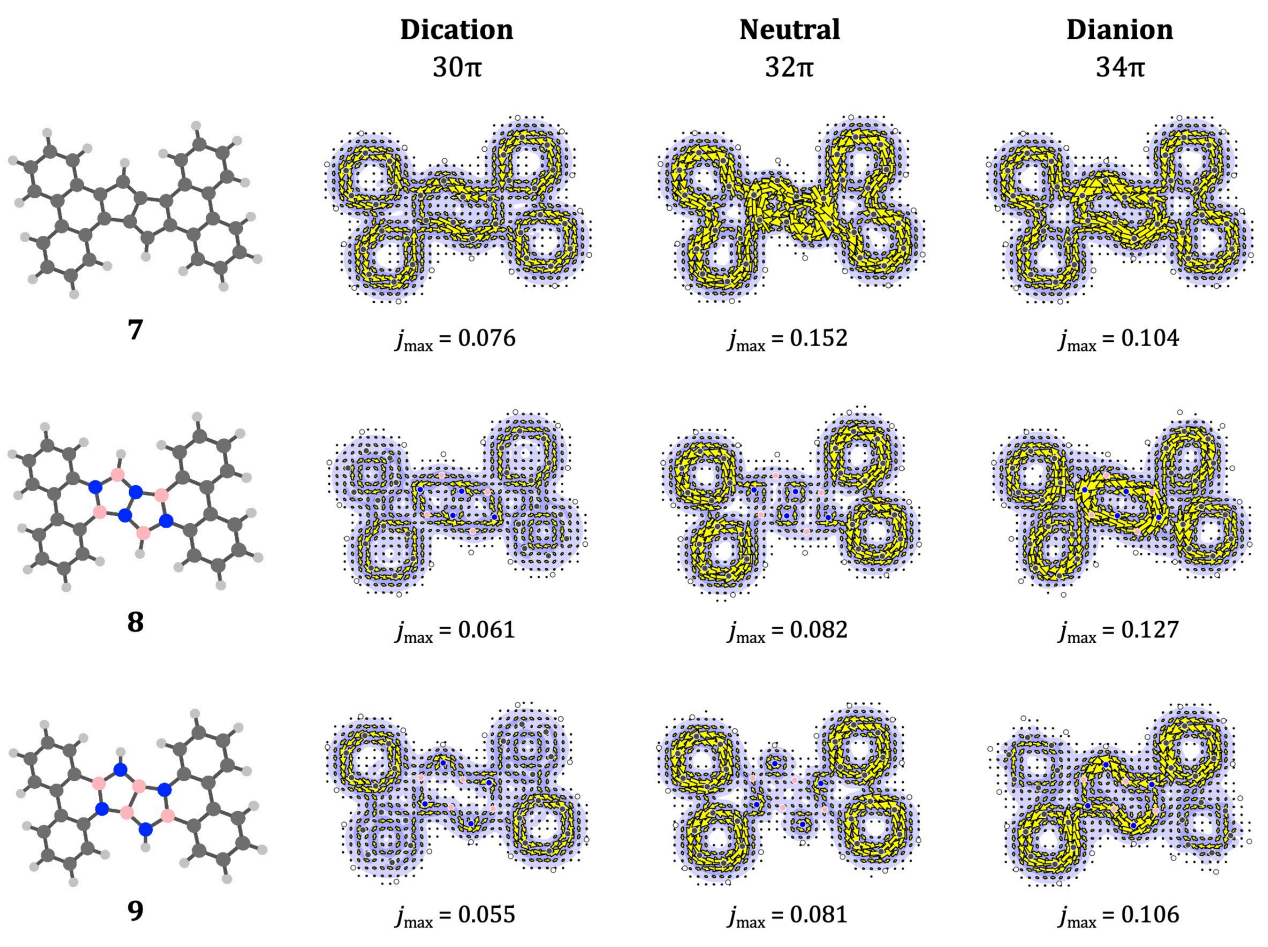
π density maps for the three phenanthrene‐extended pentalene isosteres, **7**, **8** and **9**. The maps for the dicationic, neutral and dianionic charge states, i. e. 30π , 32π and 34π systems, are shown. All maps are computed at the ($C_{2h}$ ) minimum‐energy structure of the respective neutral isostere. Plotting, colouring conventions and definition of *j* 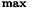 are as in previous figures.

We now turn to the larger π ‐extended systems **7–9** (Figure 8), where the formal clamping group is phenanthrene. One (**7**) seen in the synthetic work of Kashida *et al*.^[41]^. As with the naphthalene clamps, the insertion of pentalene leads to a system in which the native current of the motif is retained in all three charge states. Currents on the clamp itself show a tendency to split into local currents in the terminal hexagonal rings, as might be expected from the asymmetry in Pauling bond orders of the central ring of phenanthrene. With this proviso, the pattern for the all‐carbon **7** can be thought of as simple sum of expected currents. In this case phenanthrene is acting as a benign clamp in terms of its effect on the motif current.

The phenanthrene‐clamped heteropentalene systems show the same separation of clamp and motif currents, with the NN system **8** being weakly diatropic (**8** 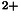 ), localised (**8** 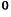 ) and strongly diatropic (**8** 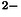 ), respectively. In the case of the neutral system **8** 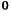 , the phenanthrene perimeter current is disturbed, with two diatropic 6‐cycle currents emerging. The dication, **8** 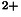 , shows elimination of one of the two currents in the clamp. Similar comments apply to the more muted currents characteristic of the BB‐motif. Finally, for the dianion, **8** 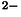 , a similar disruption is seen, but with the currents on the clamp separated into one diatropic and one paratropic current. This is not seen for the BB dianionic system, **9** 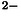 . We note that the creation of locally delocalised 6‐cycle currents in a dianionic π ‐extended boraphenalene has recently been modelled in a study by Ikeno *et al*.^[64]^

These examples illustrate the wide variability of magnetic response properties with motif, heteroatom arrangement, and clamping group. Splicing separate π systems can lead to a variety of outcomes, from preservation of the distinct aromatic signatures of the bare systems, to formation of new extended currents. The general principles for rationalisation are clear, but the balance of effects can be delicate.

## 3 Conclusions

The ipsocentric approach has been applied to calculation of current maps for three model systems (the bare (hetero)pentalenes) and used to rationalise the variability in magnetotropicity with atomic make‐up, substitution pattern and charge. This revealed a particular sensitivity of the NN‐bridged species (**2**) that has featured in synthetic work.[^39^, ^40^, ^41^] The ipsocentric approach has been central to understanding this sensitivity, in that it produces selection rules that are based on robust orbital contributions. In using systems **1** to **3** as models it is important to be aware that the (di)anionic versions of the heteropentalenes are metastable to electron loss. This difficulty is circumvented by incorporation into larger systems. Exploratory studies of larger clamped systems indicated that knowledge of magnetic response gained from the bare motifs transfers to the large systems, but the nature of the interaction of the 2 parent π systems is heavily dependent on charge in the case of the heteropentalenes.

Perhaps the most interesting system to emerge from this study is the NN‐bridged pentalene, which is as intriguing theoretically as it has proven to be accessible experimentally, and has promise as a motif for a redox‐switchable aromatic.[^4^, ^27^, ^63^]

## Conflict of Interests

The authors declare no conflict of interest.

4

## Supporting information

As a service to our authors and readers, this journal provides supporting information supplied by the authors. Such materials are peer reviewed and may be re‐organized for online delivery, but are not copy‐edited or typeset. Technical support issues arising from supporting information (other than missing files) should be addressed to the authors.

Supporting Information

Supporting Information

## Data Availability

The data that support the findings of this study are available in the supplementary material of this article.

## Footnotes

n1 In Aihara's calculation,^[33]^ bond currents in pentalene are reported as resultants of cycle currents ($j_{8}$ on the 8‐atom perimeter and $j_{5}$ in each 5‐cycle). If - is diatropic and + paratropic, Hückel‐London ring currents in the three charge states are then: j (**1**  )=$j_{8}+j_{5}$ = -2.548+1.724 =-0.824 , j (**1**  ) =$j_{8}+j_{5}$ = +2.544-0.407 =+2.137 , j (**1**  )=$j_{8}+j_{5}$ = -0.436-0.407 =-0.843 (all in units of the standard benzene current).
