# Supplementary figures and images for: Perturbing Pentalene: Aromaticity and Antiaromaticity in a Non‐Alternant Polycyclic Aromatic Hydrocarbon and BN‐Heteroanalogues
Source: Chemphyschem. 2025 Mar 16;26(8):e202401069. doi: 10.1002/cphc.202401069 (PMC12005133; doi:10.1002/cphc.202401069)

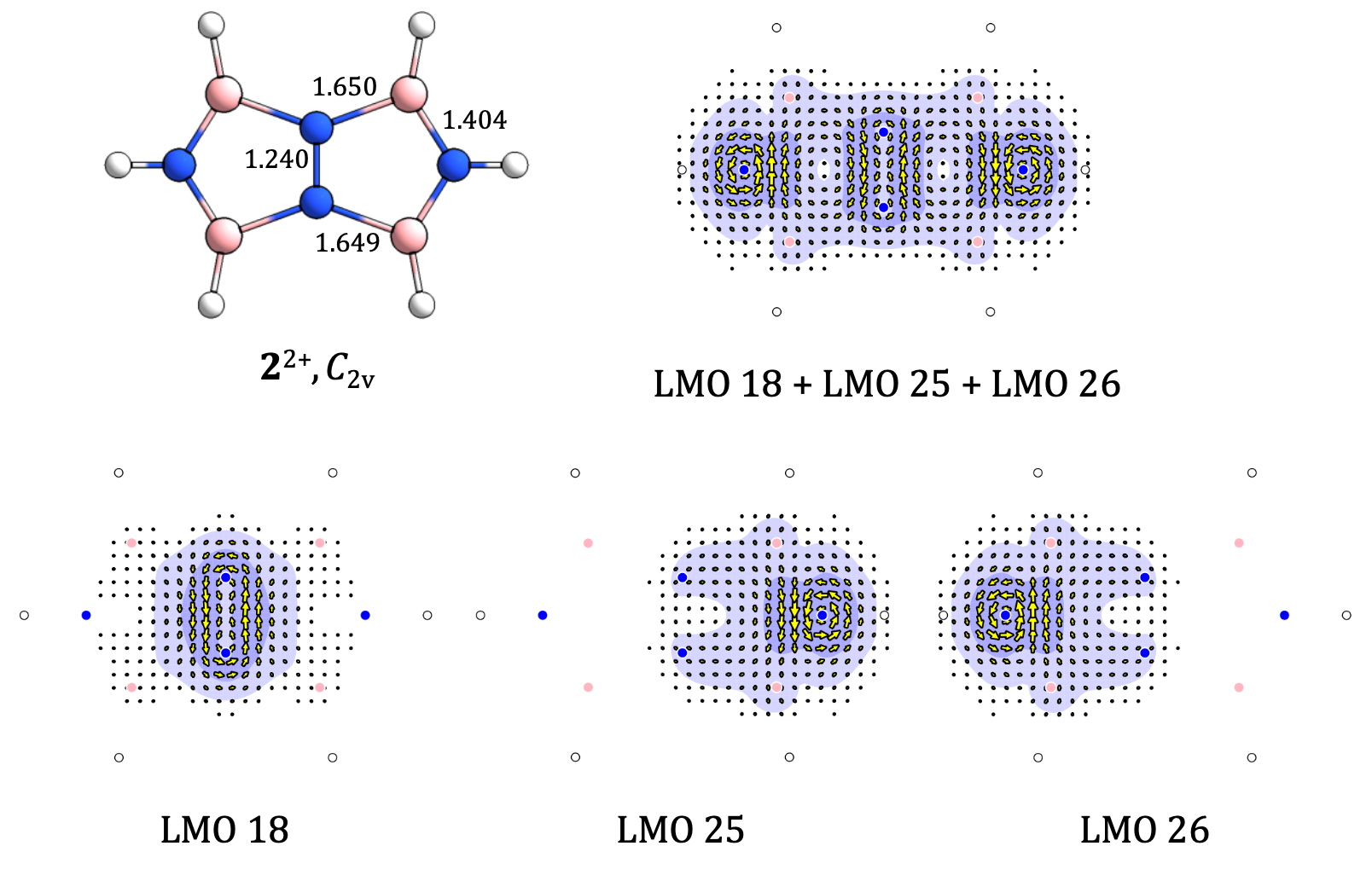

Supplement: Supplementary file 1 — Supporting Information [file CPHC-26-e202401069-s001.png]
