# Supplementary material for: Perturbing Pentalene: Aromaticity and Antiaromaticity in a Non‐Alternant Polycyclic Aromatic Hydrocarbon and BN‐Heteroanalogues
Source: Chemphyschem. 2025 Mar 16;26(8):e202401069. doi: 10.1002/cphc.202401069 (PMC12005133; doi:10.1002/cphc.202401069)
Supplement: Supplementary file 2 — Supporting Information [file CPHC-26-e202401069-s002.pdf]

ChemPhysChem

Supporting Information

Perturbing Pentalene: Aromaticity and Antiaromaticity in a Non-Alternant Polycyclic Aromatic Hydrocarbon and BN-Heteroanalogues

Cate S. Anstöter* and Patrick W. Fowler*

Supporting Information: Perturbing Pentalene: Aromaticity and Antiaromaticity in a Non-alternant Polycyclic Aromatic Hydrocarbon and its BN-heteroanalogues

Cate S. Anstöter*,† and P. W. Fowler*,‡

†*EaStCHEM School of Chemistry, Edinburgh, EH9 5FJ*

‡*School of Mathematical and Physical Science, Sheffield, S3 7HF*

E-mail: canstote@ed.ac.uk; p.w.fowler@sheffield.ac.uk

Contents

1 Supplementary Ring Current maps **S3**

1.1 Localised vs canonical orbitals: bare pentalenes S3

1.2 Relaxed vs frozen geometries: bare pentalenes S5

1.3 σ ring currents: extended pentalenes S6

1.4 Localised vs canonical orbitals: extended pentalenes S7

2 Cartesian Coordinates **S9**

2.1 Heteropentalenes S9

2.2 Extended pentalenes S15

1 Supplementary Ring Current maps

1.1 Localised vs canonical orbitals: bare pentalenes

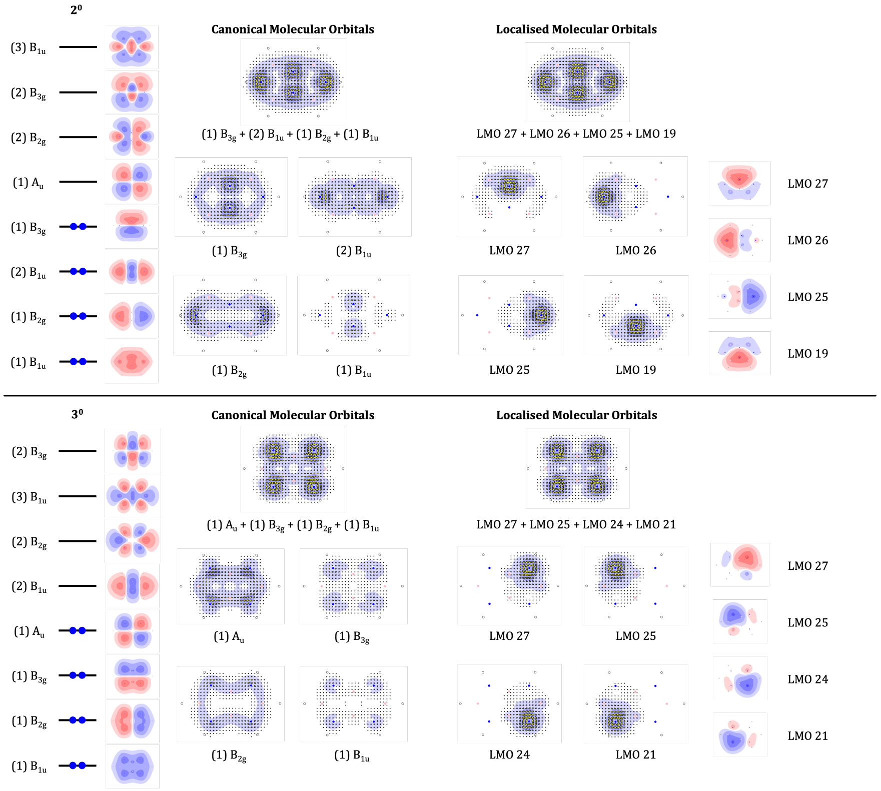

Figure S1: Comparison of canonical and localised molecular orbital accounts of π-currents in species $\mathbf{2}^0$ and $\mathbf{3}^0$. In each panel the canonical orbitals (CMO) and energies are shown on the left, together with the maps of significant CMO contributions and their combined sum. On the right are shown the significant localised contributions (LMO) and their sum, together with orbital plots. For both species, the total π map is accounted for satisfactorily by 4 molecular orbitals, either canonical or localised. The LMO picture has a straightforward interpretation in terms of nitrogen lone pairs. LMO sequence numbers refer to our implementation of the Pipek-Mezey algorithm and are essentially placeholders.

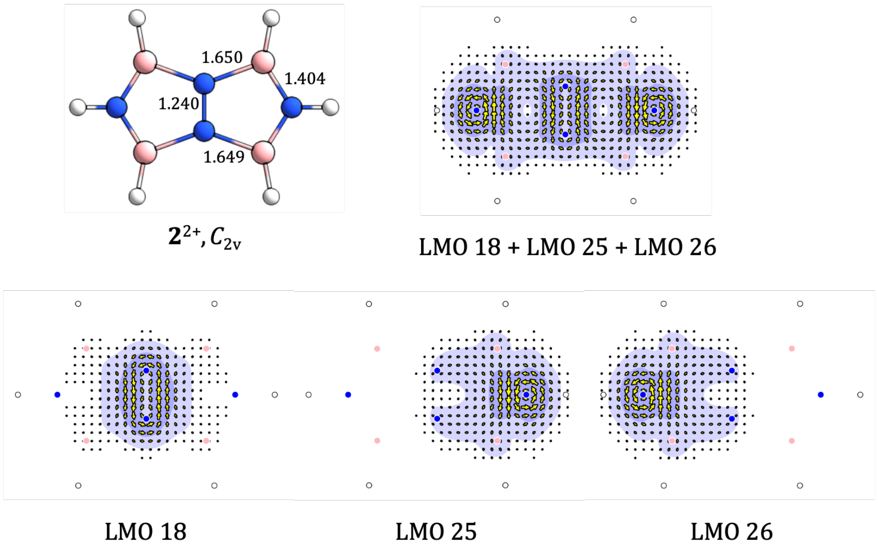

Figure S2: A schematic showing the relaxed C_{2v} geometry of the bare NN-pentalene dication, $\mathbf{2}^{2+}$, with the critical bond lengths indicated (top left). Plots of π current contributions from he three individual LMOs are shown on the bottom row, labelled by LMO sequence number as assigned in our implementation of the Pipek-Mezey localisation scheme. The summation of these three maps is shown at the top right.

1.2 Relaxed vs frozen geometries: bare pentalenes

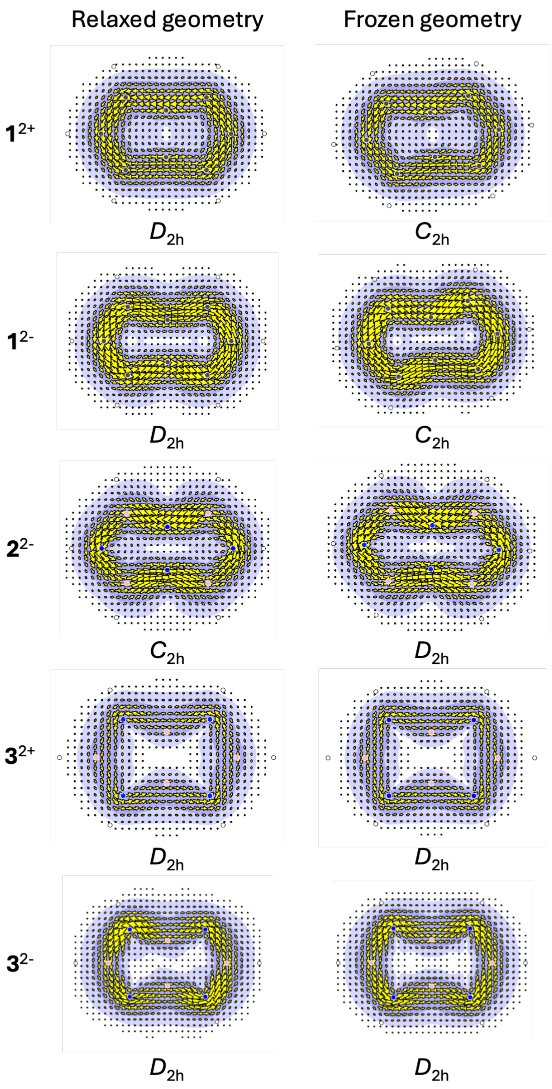

Figure S3: Total π ring-current maps are shown for all dications and anions of the bare pentalenes, **1**-**3**. The first column of maps show the ring currents in the relaxed geometry of the ion, while the second column shows the ring current in the geometry of the respective neutral species. The point-group symmetry of each structure is included below the map.

1.3 σ ring currents: extended pentalenes

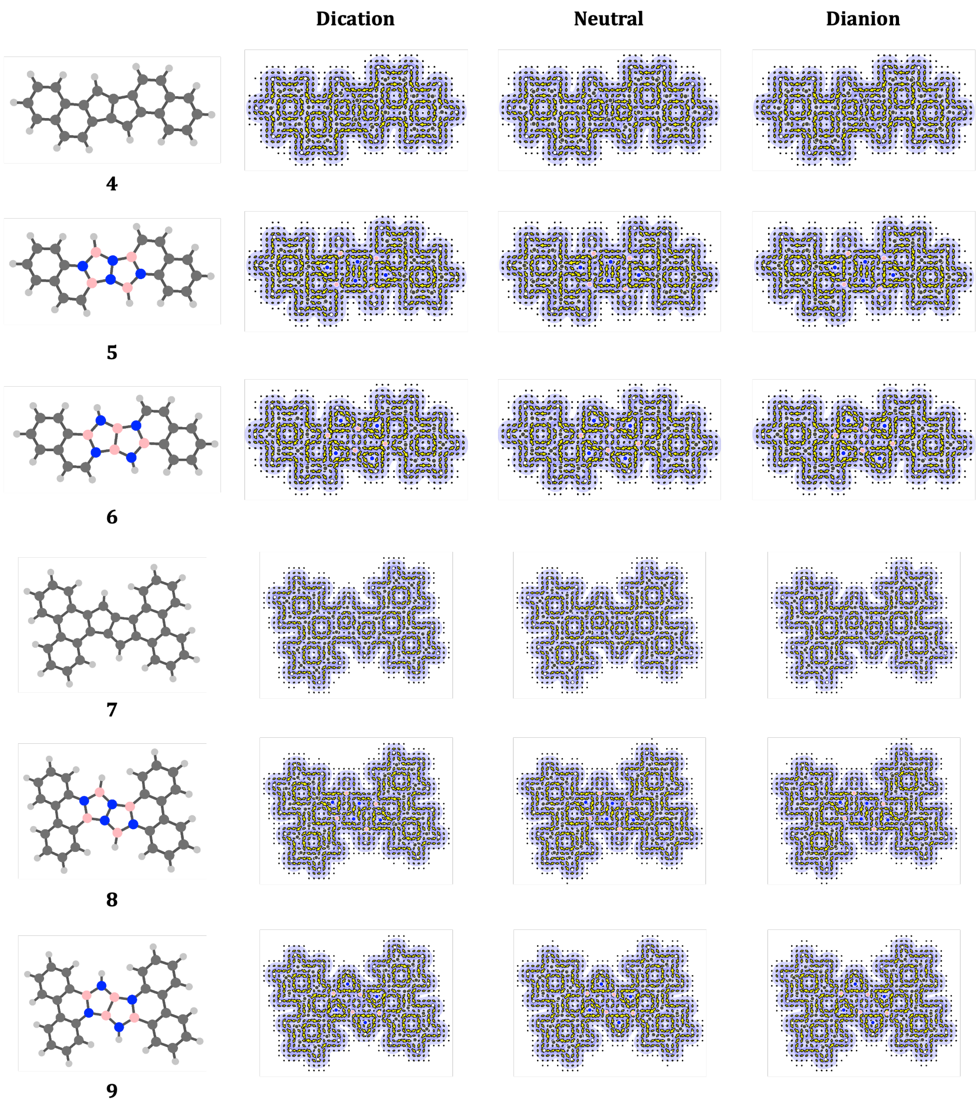

Figure S4: σ contributions to ring current maps plotted at $1a_0$ for extended systems **4-9**. Plotting conventions as in previous figures. All maps show little dependence on total charge and show localised differences between boron and nitrogen centres, but are broadly consistent with local two-electron bond circulations.

1.4 Localised vs canonical orbitals: extended pentalenes

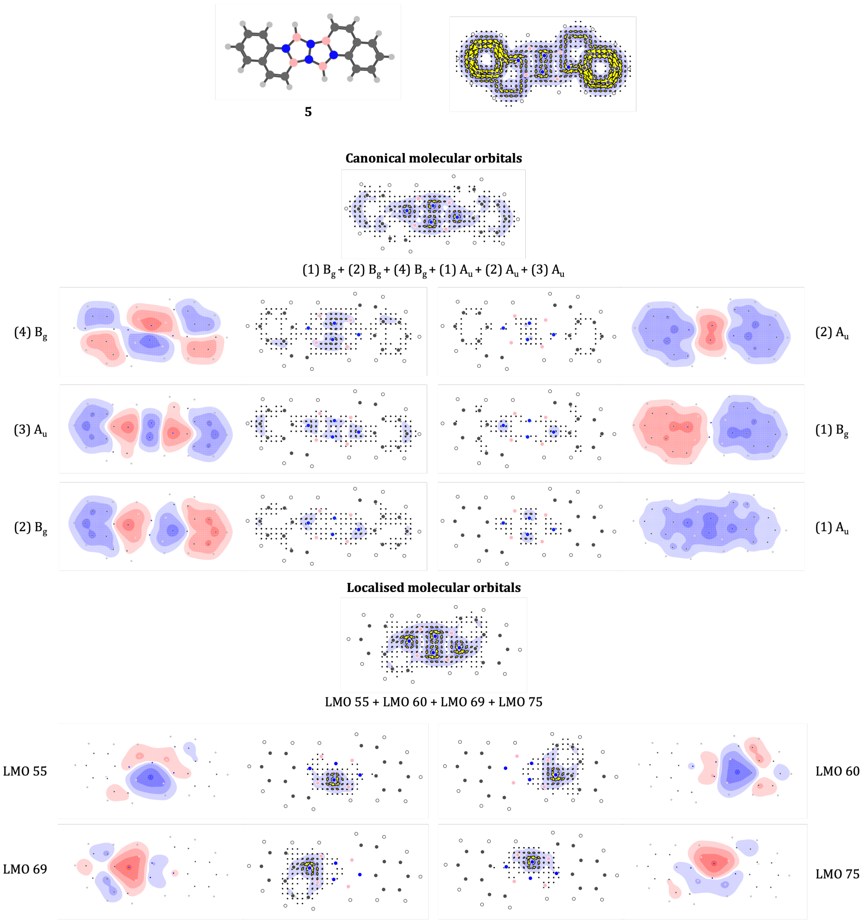

Figure S5: Analysis of orbital contributions to the π current map of species $\mathbf{5}^0$ comparing partitions into canonical and localised molecular orbitals. The map of total π current is shown at the top of the figure. Central and lower panels show the summed contributions from canonical and localised orbitals, respectively, to current in the central heteropentalene region of the framework. The localised orbitals (LMOs) give a more economical account, composed effectively of four nitrogen lone pair circulations. Canonical molecular orbitals account for essentially the same part of the current map, but only *via* a combination of six contributions spread over the four nitrogen centres.

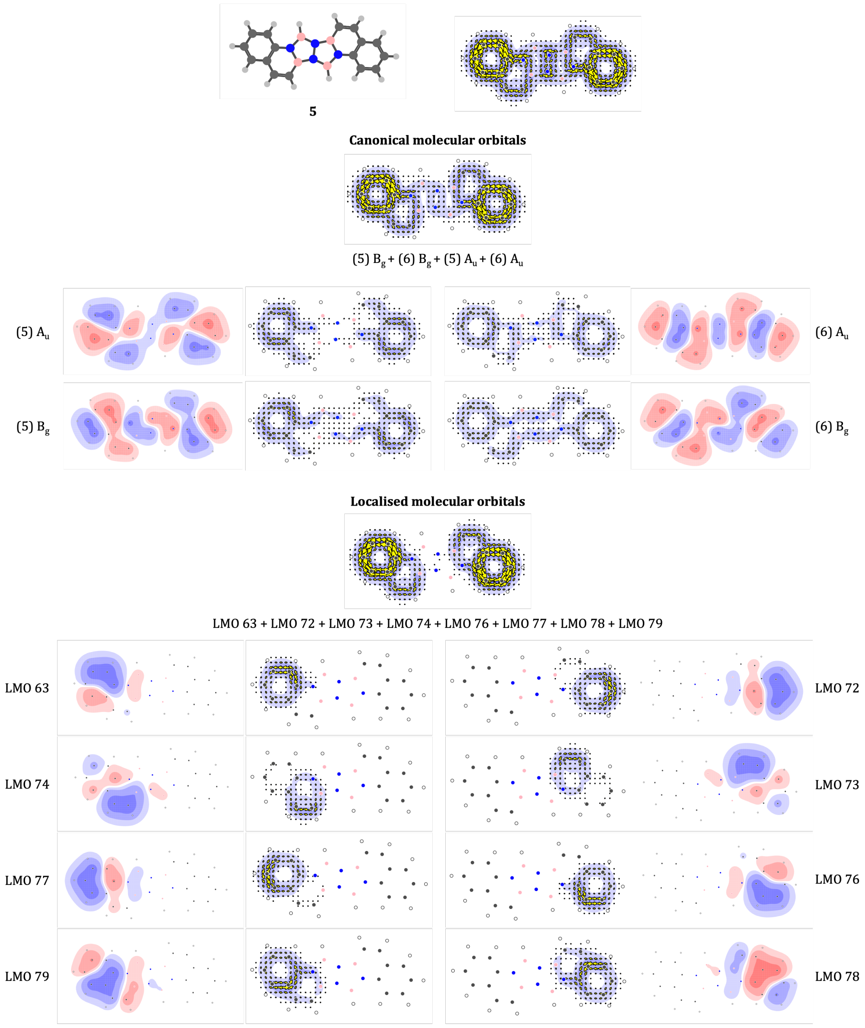

Figure S6: Analysis of orbital contributions to the π current map of species $\mathbf{5}^0$ comparing partitions into canonical and localised molecular orbitals. The map of total π current is shown at the top of the figure. Central and lower panels show the summed contributions from canonical and localised orbitals, respectively, to current in the outer 'naphthalene' regions of the framework. Here, the canonical orbitals (CMOs) give the more economical account, composed of four combinations that sum to a pair of locally delocalised ring currents on the all-carbon benzenoid rings. Localised molecular orbitals (LMOs) account for essentially the same part of the current map, but now *via* eight contributions, six of which group into two sets of three that each correspond to a archetypical locally delocalised ring current in a benzenoid ring.

2 Cartesian Coordinates

Cartesian coordinates of all optimised minimum energy geometries for systems **1-9**, for dication, neutral and dianion charge state. For all systems the point group and lowest energy mode are presented. All calculated using Q-Chem and B3LYP/6-31++G**.

2.1 Heteropentalenes

1^{2+} Pentalene Dication

B3LYP/6-31++G**

First vibrational frequency = 166.20 cm^{-1}

D_{2h}

6	4.030336837	0.000000000	0.000000000
6	2.504353376	2.256150207	0.000000000
6	0.000000000	1.456379978	0.000000000
6	0.000000000	-1.456379978	0.000000000
6	2.504353376	-2.256150207	0.000000000
6	-2.504353376	2.256150207	0.000000000
6	-4.030336837	0.000000000	0.000000000
6	-2.504353376	-2.256150207	0.000000000
1	6.088516194	0.000000000	0.000000000
1	3.234343451	4.177151974	0.000000000
1	3.234343451	-4.177151974	0.000000000
1	-3.234343451	4.177151974	0.000000000
1	-6.088516194	0.000000000	0.000000000
1	-3.234343451	-4.177151974	0.000000000

1^{0} Pentalene

B3LYP/6-31++G**

First vibrational frequency = 160.76 cm^{-1}

C_{2h}

6	2.174156254	0.060390849	0.000000000
6	1.227655447	1.220330074	0.000000000
6	-0.038192943	0.728498595	0.000000000
6	0.038192943	-0.728498595	0.000000000
6	1.466045317	-1.099070034	0.000000000
6	-1.466045317	1.099070034	0.000000000
6	-2.174156254	-0.060390849	0.000000000
6	-1.227655447	-1.220330074	0.000000000
1	3.253099799	0.156982069	0.000000000
1	1.540090121	2.259445030	0.000000000
1	1.869121258	-2.103892710	0.000000000
1	-1.869121258	2.103892710	0.000000000
1	-3.253099799	-0.156982069	0.000000000
1	-1.540090121	-2.259445030	0.000000000

1^{2-} Pentalene Dianion

B3LYP/6-31++G**

First vibrational frequency = 131.26 cm^{-1}

D_{2h}

6	0.000000000	2.188550430	0.000000000
6	1.169728180	1.367567660	0.000000000
6	0.725088300	0.000000000	0.000000000
6	-0.725088300	0.000000000	0.000000000

6	-1.169728180	1.367567660	0.000000000
6	1.169728180	-1.367567660	0.000000000
6	0.000000000	-2.188550430	0.000000000
6	-1.169728180	-1.367567660	0.000000000
1	0.000000000	3.281838330	0.000000000
1	2.198152440	1.732959010	0.000000000
1	-2.198152440	1.732959010	0.000000000
1	2.198152440	-1.732959010	0.000000000
1	0.000000000	-3.281838330	0.000000000
1	-2.198152440	-1.732959010	0.000000000

2^{2+} AKA NN-frustrated Dication

B3LYP/6-31++G**

First vibrational frequency = 153.59 cm^{-1}

C_{2v}

7	2.298690970	0.000000000	-0.000517756
5	1.548659750	0.000000000	-1.186814646
7	0.000000000	0.000000000	-0.619473766
7	0.000000000	0.000000000	0.620601604
5	1.550295960	0.000000000	1.186862364
5	-1.548659750	0.000000000	-1.186814646
7	-2.298690970	0.000000000	-0.000517756
5	-1.550295960	0.000000000	1.186862364
1	3.320467550	0.000000000	-0.000806796
1	1.759412490	0.000000000	-2.340582426
1	1.763312990	0.000000000	2.340226544
1	-1.759412490	0.000000000	-2.340582426

1	-3.320467550	0.000000000	-0.000806796
1	-1.763312989	0.000000000	2.340226545

2^0 AKA NN-frustrated Neutral

B3LYP/6-31++G**

First vibrational frequency = 138.33 cm^{-1}

D_{2h}

7	2.196052276	0.000000000	0.000000000
5	1.362271982	1.170624679	0.000000000
7	0.000000000	0.719756371	0.000000000
7	0.000000000	-0.719756371	0.000000000
5	1.362271982	-1.170624679	0.000000000
5	-1.362271982	1.170624679	0.000000000
7	-2.196052276	0.000000000	0.000000000
5	-1.362271982	-1.170624679	0.000000000
1	3.203699767	0.000000000	0.000000000
1	1.702547516	2.307267221	0.000000000
1	1.702547516	-2.307267221	0.000000000
1	-1.702547516	2.307267221	0.000000000
1	-3.203699767	0.000000000	0.000000000
1	-1.702547516	-2.307267221	0.000000000

2^{2-} AKA NN-frustrated Dianion

B3LYP/6-31++G**

First vibrational frequency = 173.87 cm^{-1}

C_{2h}

7	0.000000000	0.000000000	2.203370518
5	-1.141258123	0.230760815	1.369597236

7	-0.707973898	0.141649472	0.000000000
7	0.707973898	-0.141649472	0.000000000
5	1.141258123	-0.230760815	1.369597236
5	-1.141258123	0.230760815	-1.369597236
7	0.000000000	0.000000000	-2.203370518
5	1.141258123	-0.230760815	-1.369597236
1	0.000000000	0.000000000	3.218916000
1	-2.244503237	0.441704226	1.728567279
1	2.244503237	-0.441704226	1.728567279
1	-2.244503237	0.441704226	-1.728567279
1	0.000000000	0.000000000	-3.218916000
1	2.244503237	-0.441704226	-1.728567279

3^{2+} BB-frustrated Dication

B3LYP/6-31++G**

First vibrational frequency = 22.95 cm^{-1}

D_{2h}

5	2.243670159	0.000000000	0.000000000
7	1.385803087	1.224694116	0.000000000
5	0.000000000	0.818442215	0.000000000
5	-0.000000014	-0.818442215	0.000000000
7	1.385803087	-1.224694116	0.000000000
7	-1.385803087	1.224694116	0.000000000
5	-2.243670159	0.000000029	0.000000000
7	-1.385803087	-1.224694116	0.000000000
1	3.418830799	0.000000000	0.000000000
1	1.804097063	2.163765904	0.000000000
1	1.804097063	-2.163765904	0.000000000

1	-1.804097063	2.163765904	0.000000000
1	-3.418830799	0.000000037	0.000000000
1	-1.804097063	-2.163765904	0.000000000

3^0 BB-frustrated Neutral

B3LYP/6-31++G**

First vibrational frequency = 133.02 cm^{-1}

D_{2h}

5	2.163874900	0.000000000	0.000000000
7	1.378795000	1.233029700	0.000000000
5	0.000000000	0.831859500	0.000000000
5	0.000000000	-0.831859500	0.000000000
7	1.378795000	-1.233029700	0.000000000
7	-1.378795000	1.233029700	0.000000000
5	-2.163874900	0.000000000	0.000000000
7	-1.378795000	-1.233029700	0.000000000
1	3.359194000	0.000000000	0.000000000
1	1.805177700	2.150652000	0.000000000
1	1.805177700	-2.150652000	0.000000000
1	-1.805177700	2.150652000	0.000000000
1	-3.359194000	0.000000000	0.000000000
1	-1.805177700	-2.150652000	0.000000000

3^{2-} BB-frustrated Dianion

B3LYP/6-31++G**

First vibrational frequency = 197.37 cm^{-1}

D_{2h}

5	2.168672702	0.000000000	0.000000000
7	1.382203071	1.230297378	0.000000000
5	0.000000001	0.831146798	0.000000000
5	-0.000000001	-0.831146798	0.000000000
7	1.382203071	-1.230297378	0.000000000
7	-1.382203071	1.230297378	0.000000000
5	-2.168672702	-0.000000188	0.000000000
7	-1.382203071	-1.230297378	0.000000000
1	3.353564762	0.000000000	0.000000000
1	1.802459441	2.151623338	0.000000000
1	1.802459441	-2.151623338	0.000000000
1	-1.802459441	2.151623338	0.000000000
1	-3.353564762	-0.000000919	0.000000000
1	-1.802459441	-2.151623338	0.000000000

2.2 Extended pentalenes

4^{2+} Naphth-Pentalene Dication

B3LYP/6-31++G**

First vibrational frequency = 25.49 cm^{-1}

C_{2h}

6	2.144364212	0.299317037	0.000000000
6	1.151328178	1.345295204	0.000000000
6	-0.090031450	0.746246930	0.000000000
6	0.090031450	-0.746246930	0.000000000

6	1.474613533	−1.014207840	0.000000000
6	−1.474613533	1.014207840	0.000000000
6	−2.144364212	−0.299317037	0.000000000
6	−1.151328178	−1.345295204	0.000000000
1	1.361578729	2.407336127	0.000000000
6	2.217017493	−2.208090847	0.000000000
6	−2.217017493	2.208090847	0.000000000
6	−3.553304329	−0.382592808	0.000000000
1	−1.361578729	−2.407336127	0.000000000
6	3.553304329	0.382592808	0.000000000
6	3.593892493	−2.119463281	0.000000000
6	4.289758286	−0.861366470	0.000000000
6	−3.593892493	2.119463281	0.000000000
6	−4.289758286	0.861366470	0.000000000
6	4.265486140	1.613199231	0.000000000
6	5.652156802	1.621737704	0.000000000
6	6.364470653	0.408741742	0.000000000
6	5.691079340	−0.818656473	0.000000000
6	−5.691079340	0.818656473	0.000000000
6	−6.364470653	−0.408741742	0.000000000
6	−5.652156802	−1.621737704	0.000000000
6	−4.265486140	−1.613199231	0.000000000
1	1.723924344	−3.173840928	0.000000000
1	−1.723924344	3.173840928	0.000000000
1	4.188771222	−3.028492264	0.000000000
1	−4.188771222	3.028492264	0.000000000
1	3.725940147	2.554480124	0.000000000

1	6.189581749	2.564108929	0.000000000
1	7.450023741	0.422875749	0.000000000
1	6.258102531	-1.744483092	0.000000000
1	-6.258102531	1.744483092	0.000000000
1	-7.450023741	-0.422875749	0.000000000
1	-6.189581749	-2.564108929	0.000000000
1	-3.725940147	-2.554480124	0.000000000

4^0 Naphth-Pentalene

B3LYP/6-31++G**

First vibrational frequency = 24.01 cm^{-1}

C_{2h}

C	2.16818691	0.25371459	-0.00000000
C	1.14095415	1.32006225	-0.00000000
C	-0.08363584	0.72809523	-0.00000000
C	0.08363584	-0.72809523	-0.00000000
C	1.52428409	-1.00173381	-0.00000000
C	-1.52428409	1.00173381	-0.00000000
C	-2.16818691	-0.25371459	-0.00000000
C	-1.14095415	-1.32006225	-0.00000000
H	1.35439354	2.38258353	-0.00000000
C	2.26447687	-2.19434431	-0.00000000
C	-2.26447687	2.19434431	0.00000000
C	-3.58107736	-0.34829204	-0.00000000
H	-1.35439354	-2.38258353	-0.00000000
C	3.58107736	0.34829204	-0.00000000
C	3.64908594	-2.12779842	-0.00000000
C	4.33315905	-0.88361005	0.00000000

C	-3.64908594	2.12779842	0.00000000
C	-4.33315905	0.88361005	0.00000000
C	4.29236528	1.58308104	0.00000000
C	5.66910424	1.60663167	0.00000000
C	6.41015500	0.39560264	0.00000000
C	5.75502047	-0.81546627	0.00000000
C	-5.75502047	0.81546627	0.00000000
C	-6.41015500	-0.39560264	0.00000000
C	-5.66910424	-1.60663167	0.00000000
C	-4.29236528	-1.58308104	-0.00000000
H	1.76171047	-3.15720267	-0.00000000
H	-1.76171047	3.15720267	0.00000000
H	4.23630538	-3.04233044	0.00000000
H	-4.23630538	3.04233044	0.00000000
H	3.73674366	2.51604332	-0.00000000
H	6.19467471	2.55725263	0.00000000
H	7.49565529	0.42906947	0.00000000
H	6.32002676	-1.74428709	0.00000000
H	-6.32002676	1.74428709	0.00000000
H	-7.49565529	-0.42906947	0.00000000
H	-6.19467471	-2.55725263	0.00000000
H	-3.73674366	-2.51604332	-0.00000000

4^{2-} Naphth-Pentalene Dianion

B3LYP/6-31++G**

First vibrational frequency = 25.55 cm^{-1}

C_{2h}

6	2.182738494	0.263691222	0.000000000

6	1.235776794	1.310276525	0.000000000
6	−0.064860282	0.716353745	0.000000000
6	0.064860282	−0.716353745	0.000000000
6	1.463143507	−1.023393771	0.000000000
6	−1.463143507	1.023393771	0.000000000
6	−2.182738494	−0.263691222	0.000000000
6	−1.235776794	−1.310276525	0.000000000
1	1.459639203	2.373214848	0.000000000
6	2.186648932	−2.220524863	0.000000000
6	−2.186648932	2.220524863	0.000000000
6	−3.620970855	−0.262975522	0.000000000
1	−1.459639203	−2.373214848	0.000000000
6	3.620970855	0.262975522	0.000000000
6	3.583888860	−2.231875816	0.000000000
6	4.325335932	−1.010169037	0.000000000
6	−3.583888860	2.231875816	0.000000000
6	−4.325335932	1.010169037	0.000000000
6	4.401978616	1.446377968	0.000000000
6	5.794245258	1.428350381	0.000000000
6	6.477686988	0.192049850	0.000000000
6	5.744677194	−0.991038538	0.000000000
6	−5.744677194	0.991038538	0.000000000
6	−6.477686988	−0.192049850	0.000000000
6	−5.794245258	−1.428350381	0.000000000
6	−4.401978616	−1.446377968	0.000000000
1	1.648396818	−3.170186287	0.000000000
1	−1.648396818	3.170186287	0.000000000

1	4.129765355	-3.175230374	0.000000000
1	-4.129765355	3.175230374	0.000000000
1	3.882552038	2.402775988	0.000000000
1	6.351875684	2.364499286	0.000000000
1	7.567014817	0.163294592	0.000000000
1	6.268726433	-1.948300130	0.000000000
1	-6.268726433	1.948300130	0.000000000
1	-7.567014817	-0.163294592	0.000000000
1	-6.351875684	-2.364499286	0.000000000
1	-3.882552038	-2.402775988	0.000000000

5^{2+} Naphth-NN-frustrated Dication

B3LYP/6-31++G**

First vibrational frequency = 23.50 cm^{-1}

C_{2h}

7	2.189249611	-0.312116490	0.000000000
5	1.488492570	1.002867012	0.000000000
7	0.089657787	0.729656636	0.000000000
7	-0.089657787	-0.729656636	0.000000000
5	1.173075922	-1.377289798	0.000000000
5	-1.173075922	1.377289798	0.000000000
7	-2.189249611	0.312116490	0.000000000
5	-1.488492570	-1.002867012	0.000000000
6	3.542720233	-0.388235001	0.000000000
6	2.301363853	2.293750956	0.000000000
1	1.337339598	-2.542834303	0.000000000
1	-1.337339598	2.542834303	0.000000000

6	-3.542720233	0.388235001	0.000000000
6	-2.301363853	-2.293750956	0.000000000
6	-4.311818111	-0.861830695	0.000000000
6	-3.658541496	-2.149746245	0.000000000
6	-4.227909479	1.645286241	0.000000000
6	-5.598350141	1.677514626	0.000000000
6	-6.349330574	0.462586073	0.000000000
6	-5.709264199	-0.774324774	0.000000000
1	-1.874859862	-3.291981670	0.000000000
1	-4.311332993	-3.019784203	0.000000000
1	-3.655460389	2.565157670	0.000000000
1	-6.122170194	2.627652880	0.000000000
1	-7.434129542	0.508113554	0.000000000
1	-6.297279828	-1.686620973	0.000000000
6	3.658541496	2.149746245	0.000000000
6	4.311818111	0.861830695	0.000000000
6	5.709264199	0.774324774	0.000000000
6	6.349330574	-0.462586073	0.000000000
6	5.598350141	-1.677514626	0.000000000
6	4.227909479	-1.645286241	0.000000000
1	1.874859862	3.291981670	0.000000000
1	4.311332993	3.019784203	0.000000000
1	6.297279828	1.686620973	0.000000000
1	7.434129542	-0.508113554	0.000000000
1	6.122170194	-2.627652880	0.000000000
1	3.655460389	-2.565157670	0.000000000

5^0 Naphth-NN frustrated

B3LYP/6-31++G**

First vibrational frequency = 20.92 cm^{-1}

C_{2h}

7	2.197713110	-0.288562051	0.000000000
5	1.502954251	0.990364609	0.000000000
7	0.083489741	0.713704300	0.000000000
7	-0.083489741	-0.713704300	0.000000000
5	1.194270759	-1.339325071	0.000000000
5	-1.194270759	1.339325071	0.000000000
7	-2.197713110	0.288562051	0.000000000
5	-1.502954251	-0.990364609	0.000000000
6	3.593173890	-0.363397672	0.000000000
6	2.308072082	2.279511159	0.000000000
1	1.361077648	-2.511977941	0.000000000
1	-1.361077648	2.511977941	0.000000000
6	-3.593173890	0.363397672	0.000000000
6	-2.308072082	-2.279511159	0.000000000
6	-4.336538271	-0.851250397	0.000000000
6	-3.663608271	-2.137353938	0.000000000
6	-4.277403319	1.592332443	0.000000000
6	-5.666707489	1.626630034	0.000000000
6	-6.411109690	0.435968704	0.000000000
6	-5.745923211	-0.781373146	0.000000000
1	-1.882948202	-3.279492629	0.000000000
1	-4.313427192	-3.012633557	0.000000000
1	-3.712547468	2.518086242	0.000000000
1	-6.176388128	2.585803864	0.000000000

1	-7.496155600	0.468147915	0.000000000
1	-6.310150261	-1.710691186	0.000000000
6	3.663608271	2.137353938	0.000000000
6	4.336538271	0.851250397	0.000000000
6	5.745923211	0.781373146	0.000000000
6	6.411109690	-0.435968704	0.000000000
6	5.666707489	-1.626630034	0.000000000
6	4.277403319	-1.592332443	0.000000000
1	1.882948202	3.279492629	0.000000000
1	4.313427192	3.012633557	0.000000000
1	6.310150261	1.710691186	0.000000000
1	7.496155600	-0.468147915	0.000000000
1	6.176388128	-2.585803864	0.000000000
1	3.712547468	-2.518086242	0.000000000

5^{2-} Naphth-NN frustrated Dianion

B3LYP/6-31++G**

First vibrational frequency = 23.65 cm^{-1}

C_2

7	2.208078420	-0.297351928	-0.020472442
5	1.506309304	1.005689059	-0.021469343
7	0.081638595	0.715424399	-0.026409290
7	-0.081638595	-0.715424399	-0.026409290
5	1.207758164	-1.333848423	-0.023906254
5	-1.207758164	1.333848423	-0.023906254
7	-2.208078420	0.297351928	-0.020472442
5	-1.506309304	-1.005689059	-0.021469343

6	3.612473981	−0.359049973	−0.004861825
6	2.307393154	2.272599113	−0.020931553
1	1.369239879	−2.514081324	−0.006867638
1	−1.369239879	2.514081324	−0.006867638
6	−3.612473981	0.359049973	−0.004861825
6	−2.307393154	−2.272599113	−0.020931553
6	−4.360264927	−0.874840498	−0.001432266
6	−3.697283501	−2.142037584	−0.015865626
6	−4.300905958	1.579966305	0.002073189
6	−5.700302088	1.638271982	0.013118751
6	−6.442252889	0.443272149	0.050056045
6	−5.780244734	−0.775622752	0.052555587
1	−1.880714861	−3.274584032	−0.016847071
1	−4.340606557	−3.024196255	0.006291969
1	−3.726092964	2.501523567	−0.011557708
1	−6.206068773	2.602013527	−0.004292392
1	−7.532259070	0.473449008	0.065441617
1	−6.355745980	−1.701139035	0.074597323
6	3.697283501	2.142037584	−0.015865626
6	4.360264927	0.874840498	−0.001432266
6	5.780244734	0.775622752	0.052555587
6	6.442252889	−0.443272149	0.050056045
6	5.700302088	−1.638271982	0.013118751
6	4.300905958	−1.579966305	0.002073189
1	1.880714861	3.274584032	−0.016847071
1	4.340606557	3.024196255	0.006291969
1	6.355745980	1.701139035	0.074597323

1	7.532259070	-0.473449008	0.065441617
1	6.206068773	-2.602013527	-0.004292392
1	3.726092964	-2.501523567	-0.011557708

6^{2+} Naphth-BB-frustrated Dication

B3LYP/6-31++G**

First vibrational frequency = 16.86 cm^{-1}

C_2

5	2.185740599	-0.277642834	0.000000000
7	1.523657548	1.084625376	0.000000000
5	0.078041832	0.818984224	0.000000000
5	-0.078041832	-0.818984224	0.000000000
7	1.225555738	-1.378173887	0.000000000
7	-1.225555738	1.378173887	0.000000000
5	-2.185740599	0.277642834	0.000000000
7	-1.523657548	-1.084625376	0.000000000
6	3.724020240	-0.307636557	0.000000000
6	2.263750884	2.211062660	0.000000000
1	1.513051597	-2.352270236	0.000000000
1	-1.513051597	2.352270236	0.000000000
6	-3.724020240	0.307636557	0.000000000
6	-2.263750884	-2.211062660	0.000000000
6	-4.404204804	-0.968199607	0.000000000
6	-3.664244422	-2.173446773	0.000000000
6	-4.503882464	1.463723815	0.000000000
6	-5.910428787	1.395160537	0.000000000
6	-6.569922245	0.154163159	0.000000000

6	-5.831085667	-1.017116417	0.000000000
1	-1.744687125	-3.165201651	0.000000000
1	-4.191186478	-3.122654545	0.000000000
1	-4.038268397	2.445051247	0.000000000
1	-6.491033036	2.312593812	0.000000000
1	-7.654287330	0.116952877	0.000000000
1	-6.330484606	-1.981467675	0.000000000
6	3.664244422	2.173446773	0.000000000
6	4.404204804	0.968199607	0.000000000
6	5.831085667	1.017116417	0.000000000
6	6.569922245	-0.154163159	0.000000000
6	5.910428787	-1.395160537	0.000000000
6	4.503882464	-1.463723815	0.000000000
1	1.744687125	3.165201651	0.000000000
1	4.191186478	3.122654545	0.000000000
1	6.330484606	1.981467675	0.000000000
1	7.654287330	-0.116952877	0.000000000
1	6.491033036	-2.312593812	0.000000000
1	4.038268397	-2.445051247	0.000000000

6^0 Naphth-BB frustrated

B3LYP/6-31++G**

First vibrational frequency = 6.70 cm^{-1}

C_{2h}

5	2.157873790	-0.318039631	0.000000000
7	1.549349950	1.032394380	0.000000000
5	0.112091500	0.818862730	0.000000000

5	-0.112091500	-0.818862730	0.000000000
7	1.178293830	-1.422870650	0.000000000
7	-1.178293830	1.422870650	0.000000000
5	-2.157873790	0.318039631	0.000000000
7	-1.549349950	-1.032394380	0.000000000
6	3.695634490	-0.393385921	0.000000000
6	2.340369871	2.171490819	0.000000000
1	1.447861699	-2.398211920	0.000000000
1	-1.447861699	2.398211920	0.000000000
6	-3.695634490	0.393385921	0.000000000
6	-2.340369871	-2.171490819	0.000000000
6	-4.411415830	-0.843200609	0.000000000
6	-3.695324581	-2.106667269	0.000000000
6	-4.430790840	1.596263891	0.000000000
6	-5.821521250	1.602646802	0.000000000
6	-6.516984530	0.382041062	0.000000000
6	-5.823402320	-0.821430308	0.000000000
1	-1.819788351	-3.124997440	0.000000000
1	-4.267519401	-3.029212879	0.000000000
1	-3.900679149	2.546392921	0.000000000
1	-6.367601829	2.541487642	0.000000000
1	-7.603685060	0.378676002	0.000000000
1	-6.369509811	-1.761663188	0.000000000
6	3.695324581	2.106667269	0.000000000
6	4.411415830	0.843200609	0.000000000
6	5.823402320	0.821430308	0.000000000
6	6.516984530	-0.382041062	0.000000000

6	5.821521250	-1.602646802	0.000000000
6	4.430790840	-1.596263891	0.000000000
1	1.819788351	3.124997440	0.000000000
1	4.267519401	3.029212879	0.000000000
1	6.369509811	1.761663188	0.000000000
1	7.603685060	-0.378676002	0.000000000
1	6.367601829	-2.541487642	0.000000000
1	3.900679149	-2.546392921	0.000000000

6^{2-} Naphth-BB frustrated Dianion

B3LYP/6-31++G**

First vibrational frequency = 24.21 cm^{-1}

C_2

B	2.18287717	-0.33930875	0.15831548
N	1.55144615	1.02272842	0.22889175
B	0.11119562	0.80255106	0.32661351
B	-0.11119562	-0.80255107	0.32661349
N	1.19885415	-1.42293186	0.22736845
N	-1.19885415	1.42293186	0.22736854
B	-2.18287718	0.33930876	0.15831554
N	-1.55144615	-1.02272843	0.22889177
C	3.70613963	-0.40526997	0.03219096
C	2.34785803	2.14671206	0.12835805
H	1.46033246	-2.39793955	0.15689450
H	-1.46033248	2.39793955	0.15689462
C	-3.70613964	0.40526998	0.03219100
C	-2.34785802	-2.14671208	0.12835804

C	-4.43348260	-0.84307893	-0.04461420
C	-3.71092533	-2.09027963	0.00490900
C	-4.47116950	1.60253465	0.00126010
C	-5.85149228	1.60481885	-0.13501774
C	-6.54843101	0.37589357	-0.24682745
C	-5.83972249	-0.81902085	-0.19282519
H	-1.83609115	-3.10815906	0.16140856
H	-4.26903914	-3.02159831	-0.07122999
H	-3.95263109	2.55886443	0.07927744
H	-6.40089152	2.54557020	-0.16730050
H	-7.63081789	0.36845797	-0.37411754
H	-6.37597186	-1.76605322	-0.27355535
C	3.71092534	2.09027961	0.00490900
C	4.43348261	0.84307892	-0.04461418
C	5.83972250	0.81902086	-0.19282513
C	6.54843102	-0.37589356	-0.24682738
C	5.85149227	-1.60481884	-0.13501773
C	4.47116949	-1.60253463	0.00126005
H	1.83609118	3.10815905	0.16140855
H	4.26903911	3.02159831	-0.07123001
H	6.37597188	1.76605323	-0.27355527
H	7.63081791	-0.36845795	-0.37411747
H	6.40089149	-2.54557020	-0.16730055
H	3.95263110	-2.55886443	0.07927729

7^{2+} Phen-Pentalene Dication

B3LYP/6-31++G**

First vibrational frequency = 10.74 cm^{-1}

C_s

C	1.96576897	-0.90561888	0.00000000
C	1.71585139	0.53544653	-0.00000000
C	0.30840282	0.68425377	-0.00000000
C	-0.30840123	-0.68425902	-0.00000000
C	0.70927463	-1.61305920	0.00000000
C	-0.70927224	1.61305486	-0.00000000
C	-1.96576762	0.90561573	0.00000000
C	-1.71585070	-0.53545042	-0.00000000
C	3.27612500	-1.42616433	0.00000000
C	2.79146456	1.46451384	-0.00000000
H	0.61446507	-2.69020206	0.00000000
H	-0.61446184	2.69019803	-0.00000000
C	-3.27612309	1.42616308	0.00000000
C	-2.79146674	-1.46451583	-0.00000000
C	-4.38081711	0.49653294	0.00000000
C	-4.13415357	-0.95197847	-0.00000000
C	-3.52291581	2.83126947	0.00000000
C	-4.81051934	3.32048329	0.00000000
C	-5.88886778	2.41348407	0.00000000
C	-5.67563116	1.03548817	0.00000000
C	-2.57022471	-2.86514485	-0.00000000
C	-5.18655406	-1.88293726	-0.00000000
H	-2.68810989	3.52313082	0.00000000
H	-4.99576537	4.38908861	0.00000000
H	-6.90702111	2.79052866	0.00000000
H	-6.54320773	0.38854268	0.00000000

C	4.13415106	0.95197865	-0.00000000
C	4.38081644	-0.49653211	0.00000000
C	5.67563107	-1.03548372	0.00000000
C	5.88887207	-2.41347843	0.00000000
C	4.81052645	-3.32048042	0.00000000
C	3.52292207	-2.83127001	0.00000000
C	2.57022036	2.86514184	-0.00000000
C	5.18654968	1.88293825	-0.00000000
H	6.54320550	-0.38853590	0.00000000
H	6.90702633	-2.79051993	0.00000000
H	4.99577532	-4.38908510	0.00000000
H	2.68811851	-3.52313372	0.00000000
C	-4.94344833	-3.25508995	-0.00000000
C	-3.62953697	-3.75301566	-0.00000000
H	-1.55627124	-3.24613949	-0.00000000
H	-6.21659023	-1.55068863	-0.00000000
H	-5.78237070	-3.94377474	-0.00000000
H	-3.45091023	-4.82288163	-0.00000000
C	4.94344250	3.25509042	-0.00000000
C	3.62953070	3.75301452	-0.00000000
H	1.55626620	3.24613428	-0.00000000
H	6.21658596	1.55069095	-0.00000000
H	5.78236388	3.94377614	-0.00000000
H	3.45090228	4.82288015	-0.00000000

7^0 Phen-Pentalene

B3LYP/6-31++G**

First vibrational frequency = 16.89 cm^{-1}

C_{2h}

C	2.00346401	-0.85507480	-0.00000000
C	1.75680357	0.51430663	-0.00000000
C	0.29439629	0.67450518	-0.00000000
C	-0.29439628	-0.67450526	-0.00000000
C	0.71270731	-1.58685313	-0.00000000
C	-0.71270729	1.58685307	-0.00000000
C	-2.00346400	0.85507475	-0.00000000
C	-1.75680357	-0.51430670	-0.00000000
C	3.33158423	-1.37974310	-0.00000000
C	2.82170426	1.46330191	-0.00000000
H	0.62585809	-2.66609700	-0.00000000
H	-0.62585805	2.66609695	-0.00000000
C	-3.33158421	1.37974309	-0.00000000
C	-2.82170430	-1.46330195	-0.00000000
C	-4.42981220	0.45738494	-0.00000000
C	-4.17131578	-0.97838280	-0.00000000
C	-3.59256076	2.77470884	0.00000000
C	-4.88498144	3.26029835	0.00000000
C	-5.96873207	2.35916606	0.00000000
C	-5.74036273	0.99438845	0.00000000
C	-2.58291983	-2.86169680	-0.00000000
C	-5.21436593	-1.93719899	0.00000000
H	-2.75801736	3.46859472	0.00000000
H	-5.06591325	4.33116010	0.00000000
H	-6.98768760	2.73482668	0.00000000
H	-6.59598258	0.32918968	0.00000000

C	4.17131575	0.97838279	0.00000000
C	4.42981219	-0.45738493	0.00000000
C	5.74036273	-0.99438838	0.00000000
C	5.96873214	-2.35916598	0.00000000
C	4.88498154	-3.26029830	0.00000000
C	3.59256084	-2.77470884	-0.00000000
C	2.58291977	2.86169675	-0.00000000
C	5.21436587	1.93719899	0.00000000
H	6.59598256	-0.32918957	0.00000000
H	6.98768768	-2.73482655	0.00000000
H	5.06591339	-4.33116004	0.00000000
H	2.75801749	-3.46859476	-0.00000000
C	-4.95521256	-3.29623367	0.00000000
C	-3.62591464	-3.76546488	-0.00000000
H	-1.55907451	-3.21799101	-0.00000000
H	-6.24755894	-1.61038789	0.00000000
H	-5.78060899	-4.00205940	0.00000000
H	-3.42429986	-4.83263152	-0.00000000
C	4.95521249	3.29623367	-0.00000000
C	3.62591455	3.76546486	-0.00000000
H	1.55907443	3.21799094	-0.00000000
H	6.24755889	1.61038791	0.00000000
H	5.78060890	4.00205942	-0.00000000
H	3.42429976	4.83263150	-0.00000000

7^{2-} Phen-Pentalene Dianion

B3LYP/6-31++G**

First vibrational frequency = 22.11 cm^{-1}

C_{2h}

6	1.593500118	-1.495660492	0.000000000
6	1.789148741	-0.050831361	0.000000000
6	0.485275163	0.537999758	0.000000000
6	-0.485275163	-0.537999757	0.000000000
6	0.208529117	-1.781457127	0.000000000
6	-0.208529117	1.781457127	0.000000000
6	-1.593500118	1.495660492	0.000000000
6	-1.789148741	0.050831361	0.000000000
6	2.728672095	-2.369630205	0.000000000
6	3.091071269	0.502758548	0.000000000
1	-0.229631298	-2.773462802	0.000000000
1	0.229631298	2.773462802	0.000000000
6	-2.728672095	2.369630206	0.000000000
6	-3.091071268	-0.502758548	0.000000000
6	-4.063721871	1.820441183	0.000000000
6	-4.253299187	0.362390915	0.000000000
6	-2.589918672	3.786290812	0.000000000
6	-3.680674187	4.639933519	0.000000000
6	-4.985002086	4.105816138	0.000000000
6	-5.150141969	2.722651578	0.000000000
6	-3.317931652	-1.916094338	0.000000000
6	-5.530496813	-0.232507140	0.000000000
1	-1.584789826	4.199779030	0.000000000
1	-3.527971704	5.718629165	0.000000000
1	-5.854674030	4.760435675	0.000000000
1	-6.164128711	2.332488647	0.000000000

6	4.253299186	-0.362390920	0.000000000
6	4.063721871	-1.820441183	0.000000000
6	5.150141970	-2.722651577	0.000000000
6	4.985002086	-4.105816138	0.000000000
6	3.680674187	-4.639933519	0.000000000
6	2.589918672	-3.786290812	0.000000000
6	3.317931653	1.916094338	0.000000000
6	5.530496813	0.232507140	0.000000000
1	6.164128711	-2.332488647	0.000000000
1	5.854674031	-4.760435675	0.000000000
1	3.527971704	-5.718629164	0.000000000
1	1.584789826	-4.199779030	0.000000000
6	-5.722271408	-1.614851060	0.000000000
6	-4.590231907	-2.457966573	0.000000000
1	-2.451144156	-2.568399202	0.000000000
1	-6.414033351	0.400619644	0.000000000
1	-6.728346937	-2.029840547	0.000000000
1	-4.715733690	-3.540571338	0.000000000
6	5.722271408	1.614851060	0.000000000
6	4.590231907	2.457966573	0.000000000
1	2.451144156	2.568399202	0.000000000
1	6.414033351	-0.400619644	0.000000000
1	6.728346938	2.029840547	0.000000000
1	4.715733690	3.540571339	0.000000000

8^{2+} Phen-NN-frustrated Dication

B3LYP/6-31++G**

First vibrational frequency = 8.36 cm^{-1}

C_1

7	1.995465074	-0.952111566	-0.000769916
5	1.730978295	0.493235059	-0.007944781
7	0.307168597	0.663179444	-0.006584489
7	-0.307169152	-0.663179883	-0.006589701
5	0.708814981	-1.654434309	-0.003121072
5	-0.708815610	1.654433880	-0.003103561
7	-1.995465429	0.952111169	-0.000758136
5	-1.730978856	-0.493235493	-0.007945067
6	3.267382344	-1.456361169	0.012078275
6	2.904808979	1.474975101	-0.016711915
1	0.522253436	-2.817431179	-0.001538853
1	-0.522253404	2.817430682	-0.001490295
6	-3.267382603	1.456360862	0.012082286
6	-2.904809646	-1.474975270	-0.016713116
6	-4.409814561	0.534096576	0.012770291
6	-4.221926294	-0.905372086	-0.008559585
6	-3.484059255	2.858394705	0.027207918
6	-4.758766628	3.367655384	0.046459048
6	-5.879813178	2.487938330	0.052653983
6	-5.699640771	1.121911961	0.036294151
6	-2.763505303	-2.864408297	-0.036063932
6	-5.340126458	-1.789903462	-0.021717651
1	-2.631542832	3.525531844	0.025802831
1	-4.916607517	4.441106515	0.059364654
1	-6.883763132	2.899196382	0.070503391

1	-6.578974722	0.493057055	0.044048550
6	4.221925643	0.905371948	-0.008560696
6	4.409814090	-0.534096512	0.012763231
6	5.699640924	-1.121910607	0.036278117
6	5.879814284	-2.487936716	0.052644923
6	4.758767982	-3.367654336	0.046476325
6	3.484060133	-2.858394663	0.027225852
6	2.763505070	2.864408069	-0.036065763
6	5.340126273	1.789902801	-0.021708301
1	6.578974510	-0.493054885	0.044015196
1	6.883764672	-2.899194149	0.070482552
1	4.916609528	-4.441105139	0.059401136
1	2.631544150	-3.525532379	0.025841164
6	-5.169254966	-3.166841101	-0.041429451
6	-3.881470147	-3.714135394	-0.048497147
1	-1.773176947	-3.306372732	-0.041844723
1	-6.351339561	-1.404774103	-0.019256940
1	-6.038013590	-3.816675732	-0.052758929
1	-3.746088276	-4.790902504	-0.064577468
6	5.169255045	3.166840355	-0.041425503
6	3.881470279	3.714134796	-0.048499180
1	1.773176831	3.306372865	-0.041841362
1	6.351339264	1.404773108	-0.019229603
1	6.038013828	3.816674927	-0.052745896
1	3.746088694	4.790901932	-0.064580315

8^0 Phen-NN frustrated

B3LYP/6-31++G**

First vibrational frequency = 12.32 cm^{-1}

C_{2h}

7	2.007009875	-0.936607088	0.000000000
5	1.734702641	0.482614160	0.000000000
7	0.298721980	0.655874849	0.000000000
7	-0.298721980	-0.655874849	0.000000000
5	0.737789062	-1.633986238	0.000000000
5	-0.737789062	1.633986238	0.000000000
7	-2.007009875	0.936607088	0.000000000
5	-1.734702641	-0.482614160	0.000000000
6	3.319612403	-1.437391165	0.000000000
6	2.895467992	1.481934430	0.000000000
1	0.551799054	-2.802962338	0.000000000
1	-0.551799054	2.802962338	0.000000000
6	-3.319612403	1.437391165	0.000000000
6	-2.895467992	-1.481934430	0.000000000
6	-4.417986002	0.532960785	0.000000000
6	-4.210553087	-0.935856672	0.000000000
6	-3.548443889	2.825069052	0.000000000
6	-4.837825081	3.338867828	0.000000000
6	-5.931721468	2.465369534	0.000000000
6	-5.713325083	1.094290269	0.000000000
6	-2.723730909	-2.881120761	0.000000000
6	-5.300718821	-1.834501939	0.000000000
1	-2.699068335	3.498547244	0.000000000
1	-4.990178727	4.414146780	0.000000000

1	-6.946280096	2.851810110	0.000000000
1	-6.577689087	0.441433495	0.000000000
6	4.210553087	0.935856672	0.000000000
6	4.417986002	-0.532960785	0.000000000
6	5.713325083	-1.094290269	0.000000000
6	5.931721468	-2.465369534	0.000000000
6	4.837825081	-3.338867828	0.000000000
6	3.548443889	-2.825069052	0.000000000
6	2.723730909	2.881120761	0.000000000
6	5.300718821	1.834501939	0.000000000
1	6.577689087	-0.441433495	0.000000000
1	6.946280096	-2.851810110	0.000000000
1	4.990178727	-4.414146780	0.000000000
1	2.699068335	-3.498547244	0.000000000
6	-5.104851251	-3.210117895	0.000000000
6	-3.809852561	-3.746110994	0.000000000
1	-1.717630729	-3.288719084	0.000000000
1	-6.319564592	-1.466106447	0.000000000
1	-5.967007609	-3.871449517	0.000000000
1	-3.660048964	-4.821707941	0.000000000
6	5.104851251	3.210117895	0.000000000
6	3.809852561	3.746110994	0.000000000
1	1.717630729	3.288719084	0.000000000
1	6.319564592	1.466106447	0.000000000
1	5.967007609	3.871449517	0.000000000
1	3.660048964	4.821707941	0.000000000

8^{2-} Phen-NN frustrated Dianion

B3LYP/6-31++G**

First vibrational frequency = 9.95 cm^{-1}

C_1

7	2.010432058	-0.948224551	-0.006118621
5	1.740533097	0.495086637	-0.012448788
7	0.298853640	0.658672320	-0.005113618
7	-0.298851149	-0.658672492	-0.005086553
5	0.746251969	-1.638971671	-0.007990070
5	-0.746249443	1.638971187	-0.008045351
7	-2.010429787	0.948224782	-0.006125301
5	-1.740530727	-0.495086529	-0.012421714
6	3.329533650	-1.435011491	0.004015268
6	2.889441084	1.475310041	-0.005453064
1	0.556041823	-2.812674253	-0.030908516
1	-0.556038955	2.812672823	-0.031021835
6	-3.329531671	1.435011894	0.004015671
6	-2.889439426	-1.475309462	-0.005423547
6	-4.438164136	0.519934924	0.000897017
6	-4.233798973	-0.931403692	0.000294336
6	-3.568592512	2.819584036	0.024492793
6	-4.858716111	3.347848764	0.033152376
6	-5.953057485	2.471409401	-0.001235780
6	-5.733563093	1.100295151	-0.023277978
6	-2.737994550	-2.889471733	0.014822964
6	-5.318439282	-1.839099120	-0.019300342
1	-2.713631437	3.487776878	0.041794655

1	-5.008379578	4.425293111	0.065923735
1	-6.972264545	2.856201592	-0.005277363
1	-6.601983250	0.449770266	-0.046067752
6	4.233801433	0.931404545	0.000305273
6	4.438166805	-0.519934732	0.000905481
6	5.733563745	-1.100299511	-0.023280353
6	5.953054952	-2.471414882	-0.001251171
6	4.858712310	-3.347852610	0.033127253
6	3.568590216	-2.819584338	0.024472886
6	2.737992996	2.889472237	0.014769775
6	5.318439642	1.839104098	-0.019245730
1	6.601985240	-0.449777994	-0.046078259
1	6.972261076	-2.856210431	-0.005302768
1	5.008373839	-4.425297867	0.065885683
1	2.713627295	-3.487775058	0.041764974
6	-5.134190056	-3.216913277	-0.015270641
6	-3.819918615	-3.751319600	0.015030436
1	-1.731631096	-3.300344680	0.030596640
1	-6.339665761	-1.468203547	-0.042156983
1	-5.998658794	-3.878267661	-0.032186673
1	-3.665799573	-4.828955278	0.030178298
6	5.134187970	3.216918025	-0.015233730
6	3.819914588	3.751322675	0.015007563
1	1.731628374	3.300342841	0.030507729
1	6.339667673	1.468210478	-0.042051179
1	5.998656168	3.878274096	-0.032116619
1	3.665792791	4.828958082	0.030133961

9^{2+} Phen-BB-frustrated Dication

B3LYP/6-31++G**

First vibrational frequency = 8.82 cm^{-1}

C_{2h}

B	1.99016257	-0.91977806	0.00000000
N	1.78809948	0.56412156	0.00000000
B	0.33317758	0.75392209	-0.00000000
B	-0.33317757	-0.75392209	0.00000000
N	0.74412184	-1.68101352	0.00000000
N	-0.74412184	1.68101352	-0.00000000
B	-1.99016257	0.91977806	-0.00000000
N	-1.78809948	-0.56412156	-0.00000000
C	3.43019087	-1.44940506	0.00000000
C	2.84563896	1.43338658	0.00000000
H	0.73717815	-2.69522648	0.00000000
H	-0.73717815	2.69522647	-0.00000000
C	-3.43019087	1.44940506	-0.00000000
C	-2.84563896	-1.43338658	-0.00000000
C	-4.50252392	0.49349366	0.00000000
C	-4.21953490	-0.93043361	0.00000000
C	-3.73728034	2.81362731	-0.00000000
C	-5.06311425	3.26659244	0.00000000
C	-6.11237241	2.33768065	0.00000000
C	-5.84098160	0.97762140	0.00000000
C	-2.60411678	-2.83001669	-0.00000000
C	-5.25354847	-1.90161662	-0.00000000

H	-2.94062883	3.55220362	-0.00000000
H	-5.27619388	4.33082681	0.00000000
H	-7.14172577	2.68098005	0.00000000
H	-6.67879199	0.29293987	0.00000000
C	4.21953490	0.93043361	0.00000000
C	4.50252392	-0.49349366	0.00000000
C	5.84098160	-0.97762140	-0.00000000
C	6.11237242	-2.33768065	-0.00000000
C	5.06311425	-3.26659244	0.00000000
C	3.73728034	-2.81362731	0.00000000
C	2.60411678	2.83001669	0.00000000
C	5.25354847	1.90161662	-0.00000000
H	6.67879199	-0.29293986	-0.00000000
H	7.14172577	-2.68098005	-0.00000000
H	5.27619389	-4.33082681	0.00000000
H	2.94062883	-3.55220362	0.00000000
C	-4.98784759	-3.25386530	-0.00000000
C	-3.64502593	-3.72604096	-0.00000000
H	-1.58120903	-3.18609126	-0.00000000
H	-6.28789601	-1.58681588	-0.00000000
H	-5.80662344	-3.96573894	-0.00000000
H	-3.44955416	-4.79337992	-0.00000000
C	4.98784758	3.25386530	-0.00000000
C	3.64502593	3.72604096	0.00000000
H	1.58120903	3.18609125	0.00000000
H	6.28789601	1.58681588	-0.00000000
H	5.80662343	3.96573895	-0.00000000

H	3.44955415	4.79337992	0.00000000

9^0 Phen-BB frustrated

B3LYP/6-31++G**

First vibrational frequency = 16.37 cm^{-1}

C_{2h}

B	1.96547184	-0.92492445	0.00000000
N	1.78789617	0.53385152	0.00000000
B	0.35089684	0.74974916	0.00000000
B	-0.35089684	-0.74974916	0.00000000
N	0.71382634	-1.69977605	0.00000000
N	-0.71382634	1.69977605	0.00000000
B	-1.96547184	0.92492445	0.00000000
N	-1.78789617	-0.53385152	0.00000000
C	3.40564402	-1.47218731	0.00000000
C	2.87881592	1.41376271	0.00000000
H	0.69606512	-2.71100174	0.00000000
H	-0.69606512	2.71100174	0.00000000
C	-3.40564402	1.47218731	-0.00000000
C	-2.87881592	-1.41376271	0.00000000
C	-4.48197637	0.54002676	0.00000000
C	-4.21131316	-0.91739494	0.00000000
C	-3.68918938	2.85245941	-0.00000000
C	-4.99290242	3.33270161	-0.00000000
C	-6.05063334	2.41380992	-0.00000000
C	-5.79985019	1.04636341	-0.00000000
C	-2.64807792	-2.80143136	0.00000000
C	-5.25506641	-1.86671571	0.00000000

H	-2.86636366	3.56363361	-0.00000000
H	-5.19084841	4.40051562	-0.00000000
H	-7.07791750	2.76791611	-0.00000000
H	-6.64906222	0.37333574	-0.00000000
C	4.21131316	0.91739494	0.00000000
C	4.48197637	-0.54002676	0.00000000
C	5.79985019	-1.04636341	0.00000000
C	6.05063334	-2.41380992	-0.00000000
C	4.99290242	-3.33270161	-0.00000000
C	3.68918938	-2.85245941	-0.00000000
C	2.64807792	2.80143136	-0.00000000
C	5.25506641	1.86671571	0.00000000
H	6.64906222	-0.37333574	0.00000000
H	7.07791750	-2.76791611	-0.00000000
H	5.19084841	-4.40051562	-0.00000000
H	2.86636366	-3.56363361	-0.00000000
C	-5.01775455	-3.23586574	0.00000000
C	-3.69997156	-3.70737348	0.00000000
H	-1.62474976	-3.16078332	0.00000000
H	-6.28471973	-1.52959720	0.00000000
H	-5.85217937	-3.93038951	0.00000000
H	-3.49603254	-4.77418947	0.00000000
C	5.01775455	3.23586574	-0.00000000
C	3.69997156	3.70737348	-0.00000000
H	1.62474976	3.16078332	-0.00000000
H	6.28471973	1.52959720	0.00000000
H	5.85217937	3.93038951	-0.00000000

H	3.49603254	4.77418947	-0.00000000

9^{2-} Phen-BB frustrated Dianion

B3LYP/6-31++G**

First vibrational frequency = 24.21 cm^{-1}

C_2

5	2.209088074	-0.003706803	0.120397075
7	1.378042359	1.246578657	0.190973339
5	-0.012033581	0.810128360	0.288695110
5	0.012033581	-0.810128360	0.288695110
7	1.401133335	-1.224259867	0.189450060
7	-1.401133335	1.224259867	0.189450060
5	-2.209088074	0.003706803	0.120397075
7	-1.378042359	-1.246578657	0.190973339
6	3.724687581	0.162539656	-0.005727454
6	1.994431805	2.478518354	0.090439626
1	1.807716882	-2.148219211	0.118976112
1	-1.807716882	2.148219211	0.118976112
6	-3.724687581	-0.162539656	-0.005727454
6	-1.994431805	-2.478518354	0.090439626
6	-4.253914160	-1.506906529	-0.082532610
6	-3.350248218	-2.629843197	-0.033009399
6	-4.662745829	0.904587535	-0.036658324
6	-6.027390195	0.697121353	-0.172936172
6	-6.529516723	-0.623427846	-0.284745885
6	-5.647483030	-1.696789429	-0.230743611
1	-1.342525913	-3.351046031	0.123490180
1	-3.760379406	-3.635148170	-0.109148384

1	-4.295530718	1.928600209	0.041359013
1	-6.713347078	1.543475856	-0.205218942
1	-7.598207399	-0.795233118	-0.412035979
1	-6.033616110	-2.714303611	-0.311473765
6	3.350248218	2.629843197	-0.033009399
6	4.253914160	1.506906529	-0.082532610
6	5.647483030	1.696789429	-0.230743611
6	6.529516723	0.623427846	-0.284745885
6	6.027390195	-0.697121353	-0.172936172
6	4.662745829	-0.904587535	-0.036658324
1	1.342525913	3.351046031	0.123490180
1	3.760379406	3.635148170	-0.109148384
1	6.033616110	2.714303611	-0.311473765
1	7.598207399	0.795233118	-0.412035979
1	6.713347078	-1.543475856	-0.205218942
1	4.295530718	-1.928600209	0.041359013
